# Neonatal mortality burden and trends in UNHCR refugee camps, 2006–2017: a retrospective analysis

**DOI:** 10.1186/s12889-021-10343-5

**Published:** 2021-02-22

**Authors:** Hannah Tappis, Marwa Ramadan, Josep Vargas, Vincent Kahi, Heiko Hering, Catrin Schulte-Hillen, Paul Spiegel

**Affiliations:** 1 Center for Humanitarian Health, Johns Hopkins Bloomberg School of Public Health, 615 N. Wolfe St, Baltimore, MD 21201 USA; 2 United Nations High Commissioner for Refugees, Geneva, Switzerland

**Keywords:** Newborn health, Mortality, Stillbirth, Neonatal death, Refugee, Migration, Humanitarian, Community surveillance, Health systems

## Abstract

**Background:**

More than 100 million people were forcibly displaced over the last decade, including millions of refugees displaced across international borders. Although refugee health and well-being has gained increasing attention from researchers in recent years, few studies have examined refugee birth outcomes or newborn health on a regional or global scale. This study uses routine health information system data to examine neonatal mortality burden and trends in refugee camps between 2006 and 2017.

**Methods:**

Refugee population and mortality data were exported from the United Nations High Commissioner for Refugees (UNHCR) Health Information System (HIS) database. Tableau was used to export the data. Stata was used for data cleaning and statistical analysis. Neonatal mortality burdens and trends in refugee camps were analyzed and compared to national and subnational neonatal mortality rates captured by household surveys.

**Findings:**

One hundred fifty refugee camps in 21 countries were included in this study, with an average population of 1,725,433 between 2006 and 2017. A total of 663,892 live births and 3382 neonatal deaths were captured during this period. Annual country-level refugee camp neonatal mortality rates (NMR) ranged from 12 to 56 neonatal deaths per 1000 live births. In most countries and years where national population-based surveys are available, refugee camp NMR as reported in the UNHCR HIS was lower than that of the immediate host community.

**Conclusion:**

The UNHCR HIS provides insights into the neonatal mortality burden among refugees in camp settings and issues to consider in design and use of routine health information systems to monitor neonatal health in sub-national populations. Increased visibility of neonatal deaths and stillbirths among displaced populations can drive advocacy and inform decisions needed to strengthen health systems. Efforts to count every stillbirth and neonatal death are critical, as well as improvements to reporting systems and mechanisms for data review within broader efforts to improve the quality of neonatal care practices within and outside of health facilities.

**Supplementary Information:**

The online version contains supplementary material available at 10.1186/s12889-021-10343-5.

## Background

The day of birth and first month of life are the riskiest periods for child survival. Deaths among children aged 1 month to 5 years old have declined dramatically in recent decades, but too little progress has been made reducing the preventable deaths of newborns – which accounted for 46% of all under-5 deaths in 2017 [1]. Recent estimates suggest that as many as 7000 newborns die every day, and 2.6 million stillbirths occur every year [1]. Substantial evidence has shown that most of these deaths can be prevented [2]. The Sustainable Development Goals established at the 2015 United Nations General Assembly include explicit targets for reducing newborn mortality to as low as 12 per 1000 live births in every country by 2030 [3, 4].

The United Nations (UN) Secretary General’s *Global Strategy for Women, Children, and Adolescents’ Health 2016–2030* and global networks, including the Every Newborn Action Plan movement and Partnership for Maternal, Newborn and Child Health, recognize that these goals will not be met without urgent action in fragile and humanitarian settings [5, 6]. Many countries with the highest neonatal mortality rates are currently or have recently been affected by complex humanitarian emergencies [7, 8]. Although the widely referenced *2018 Interagency Fie*ld *Manual for Reproductive Health in Humanitarian Crises* explicitly highlights the importance of focusing on the maternal-newborn dyad, and the *Newborn Health in Humanitarian Settings Field Guide* summarizes existing evidence-based guidelines for interventions aimed at reducing neonatal mortality, limited information exists on the implementation or outcomes of these interventions in humanitarian contexts [9, 10].

Humanitarian contexts are diverse, with varying population dynamics, burden of disease, economic opportunities, and health system capacity. Across the globe, the number of individuals who have been forcibly displaced as a result of conflict, persecution or human rights violations is at an all-time high. The United Nations High Commissioner for Refugees (UNHCR) estimates that by the end of 2018, there were 70.8 million forcibly displaced people including 41.3 million people displaced within the borders of their own county, 25.9 million refugees displaced across international borders, and 3.5 million registered asylum seekers [11]. Nearly four out of five refugees live in countries neighboring their country of origin, and approximately three out of five refugees live in urban areas [11]. Globally, the majority of refugees live outside of camps. However, the living conditions and policies governing refugee accommodation, movement, livelihoods, and access to services vary substantially from country to country. There are millions of refugees who continue to live in camps, and host governments continue to create new camps in response to context-specific needs.. In some countries, such as Bangladesh, Ethiopia, Kenya, Nigeria, and Tanzania, UNHCR reports that the majority of refugees continue to live in camps [11].

Studies have repeatedly shown that health service coverage and quality in UNHCR-supported refugee camps are often higher than that in the immediately surrounding host country population [12–15]. Previous studies using UNHCR data have shown that refugees living in protracted camps generally have lower crude and under-five mortality rates than surrounding host communities, and possibly lower maternal mortality rates [16, 17]. However, little is known about if or how the newborn morality burden has changed over time. The UNHCR Health Information System (HIS), a standardized system for routine reporting and analysis of public health indicators established in 2006, provides a valuable source of data for addressing these questions [18]. A previous study, conducted as part of the 2014 *Interagency Working Group for Reproductive Health in Crisis’s Global Evaluation,* − examined reproductive health indicators, including neonatal mortality, in UNHCR post-emergency camps 2007–2013 in 10 countries [19]. We build on this study with a more extensive analysis of neonatal mortality data availability, burden and trends over the period from 2006 to 2017 to inform UNHCR strategies and global efforts to improve newborn care for conflict-affected populations.

## Methods

Data on refugee camp populations, births and deaths over the period of 2006 to 2017 were extracted from the global UNHCR HIS Database [20]. For comparison purposes, national and sub-national neonatal mortality rates (NMR) were extracted from Demographic and Health Surveys and Multiple Indicator Cluster Surveys conducted between 2006 and 2017 in countries with refugee camps using the UNHCR HIS [21, 22]. Analysis included the following variables: country, name of camp, year and month of reporting, total camp population, total number of live births, total number of neonatal deaths, NMR (number of deaths during first 28 days of life per 1000 live births), and national/sub-national host country neonatal mortality rates. Additional variables included in exploratory analyses and data quality checks included infant mortality rate (number of deaths during first year of life per 1000 live births), stillbirth rate (number of fetal deaths after 22 weeks gestation per 1000 births [livebirths and stillbirths]), and under-five mortality rate (number of deaths during first 5 years of life per 1000 live births) [20]. Stillbirth rates were not analyzed alongside NMRs due to high frequency of missing data.

**Fig. 1:**
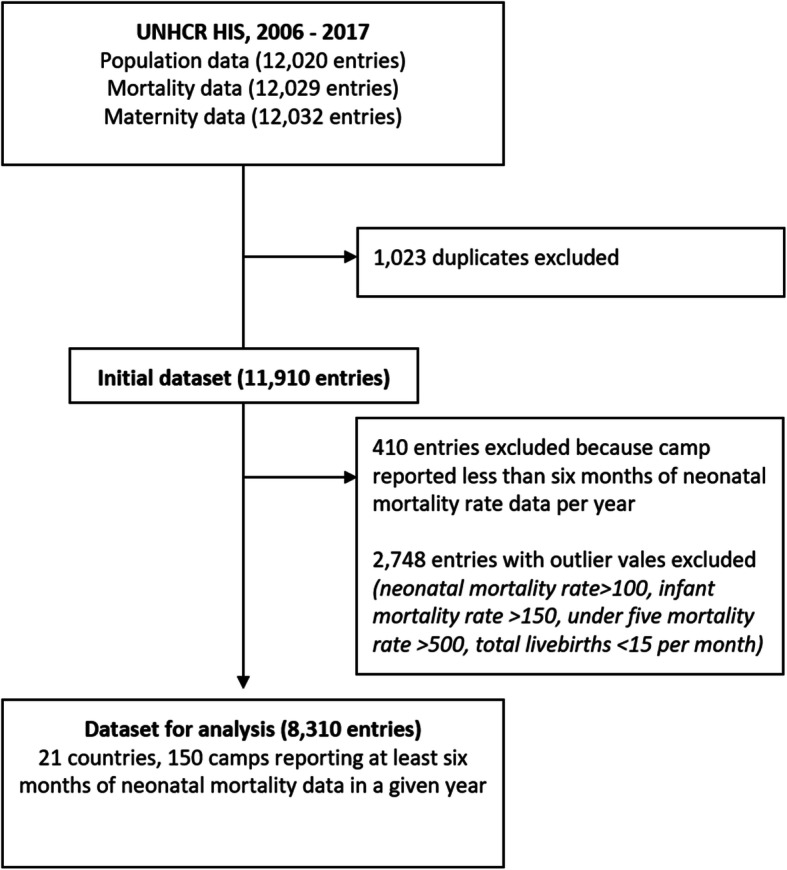
UNHCR Health Information System data included in analysis

Three UNHCR HIS datasets were merged using camp name and reporting year/month as a unique identifier: a population dataset (12,020 entries), a mortality dataset (12,029 entries) and a maternity dataset (12,032 entries), resulting in a master database with 11,910 entries after dropping of duplicates. Camps with less than 6 months of data on neonatal mortality in a given year were excluded from the analysis (410 entries). Entries with a NMR > 100, infant mortality rate > 150, under-five mortality rate > 500 or total livebirths less than 15 per month were also dropped from the analysis as fairly implausible outliers, resulting in the analytic database with 8310 entries from 150 camps in 21 countries (Fig. 1).

UNHCR implementing partners use tally sheets to aggregate data from standardized register books, and submit electronic reporting forms including the number of births and deaths recorded, on a monthly basis. Because the UNHCR HIS automatically calculates a mortality rate of 0% where the number of deaths is left blank, it is impossible to discern whether camps with a NMR of zero in a given month or year actually had zero deaths, or whether data is missing. Camps with months reporting zero newborn deaths in a given month were flagged and a sensitivity analysis conducted to calculate annual NMR with and without these months included.

Host country NMRs were matched to 947 entries in UNHCR’s camp database based on camp location and the year the Demographic and Health Survey (DHS) or Multiple Indicator Cluster survey (MICS) was conducted, with an understanding that DHS and MICS typically reports NMR for the five and 2 year periods prior to data collection, respectively.

Data cleaning and analysis was conducted between the period of December 2017 to October 2018 using Stata version 15, Tableau Prep and Tableau desktop. Analysis included calculation of median refugee camp populations, number of refugee live births per country and camp per year, NMRs in UNHCR refugee camps (including and excluding months with zero deaths reported) and visual comparison of refugee camp and host country population mortality rates in locations with both camp and host population data survey data available in at least 1 year.

## Results

The analysis included data from 150 refugee camps located in 21 countries, with an average population of 1,725,433 between 2006 and 2017. Kenya (2006–2017) had the largest median population of 84,338 across 7 camps followed by South Sudan (2013–2017; median population 39,799 across 13 camps), while Central African Republic (2013) had the lowest median population of 5987 across two camps.

Data availability varied substantially across countries and over time. Kenya, one of the first countries to adopt the UNHCR HIS in 2006, had the most consistent reporting, with 12 monthly reports per year available for the majority of camps. Thailand and Uganda also showed consistent reporting of 12 months per year for most camps starting 2008 onward. A detailed summary of data available per camp per year is provided in Supplementary File 1.

**Fig. 2:**
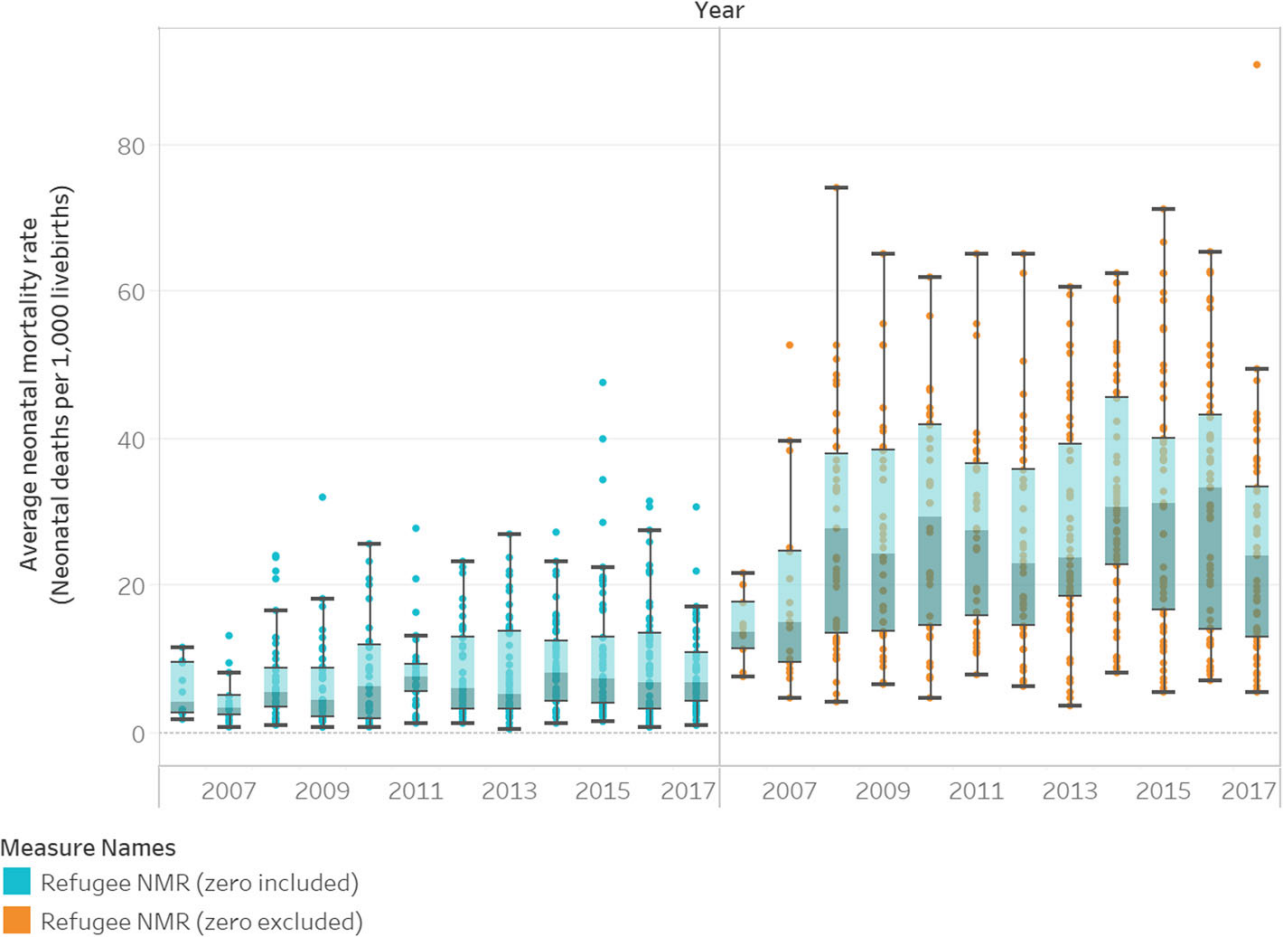
Global refugee neonatal mortality rate (NMR), with months reporting zero deaths included and excluded

Available reports documented a total of 663,892 live births and 3382 neonatal deaths in refugee camps between 2006 and 2017. Only 21% of database entries (*n* = 1733) reported neonatal deaths. Figure 2 shows the global refugee NMR for each year, 2006–2017, as reported in the UNHCR HIS (Fig. 2a), and the aggregate refugee NMR for months where newborn deaths are reported (Fig. 2b). Inclusion/exclusion of reports with zero neonatal deaths changes the magnitude of the neonatal mortality burden in refugee camps from an aggregate NMR of 5.2 deaths to 24.5 deaths per 1000 live births for the period 2006–2017, but trends in NMR over time remain similar. The analysis that follows excludes months where zero newborn deaths are reported (upper bound of NMR sensitivity analysis presented in Supplementary File 2)*,* resulting in a conservative assessment of newborn health in refugee camps.

**Fig. 3:**
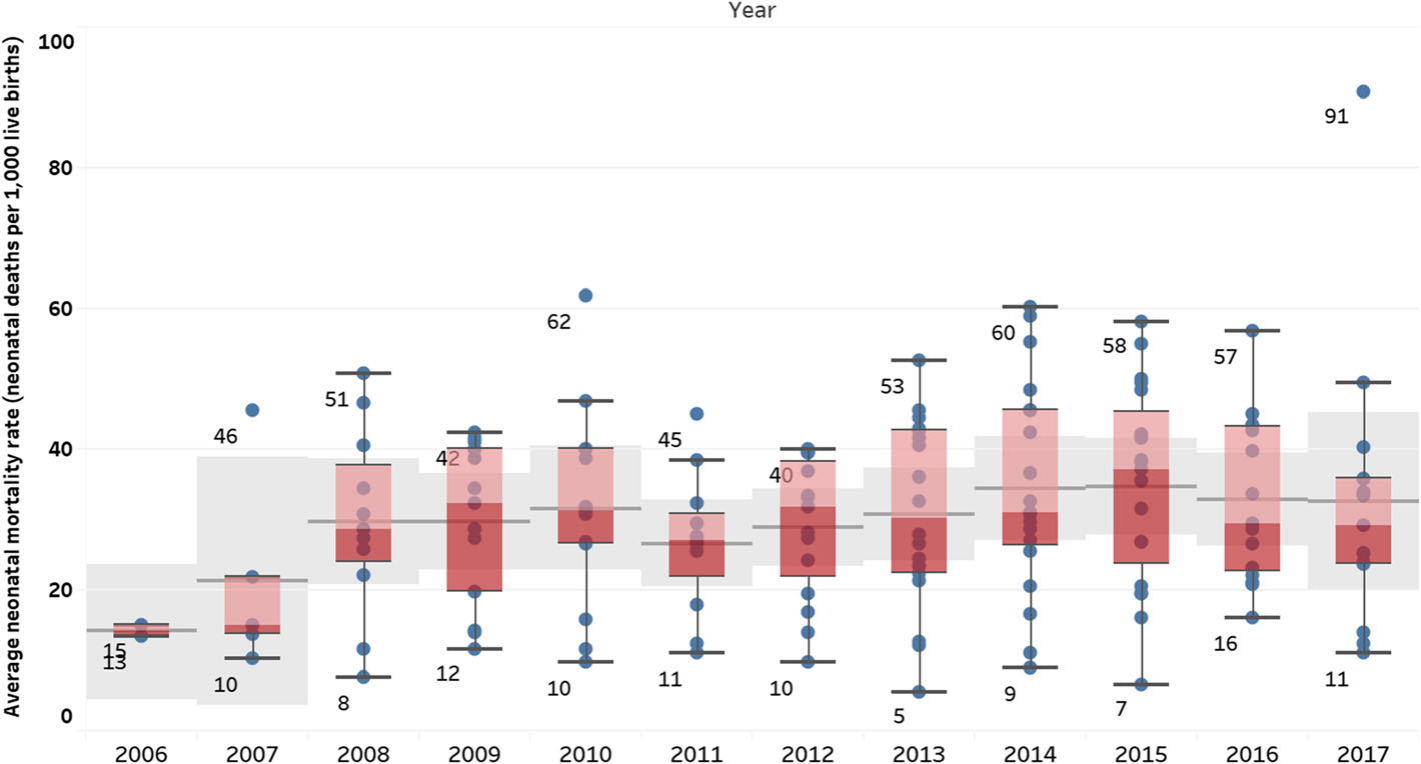
Global refugee camp NMR by year (mean / 95% confidence interval), 2006–2017, based on reports from months with one or more neonatal deaths reported in the UNHCR HIS

**Fig. 4:**
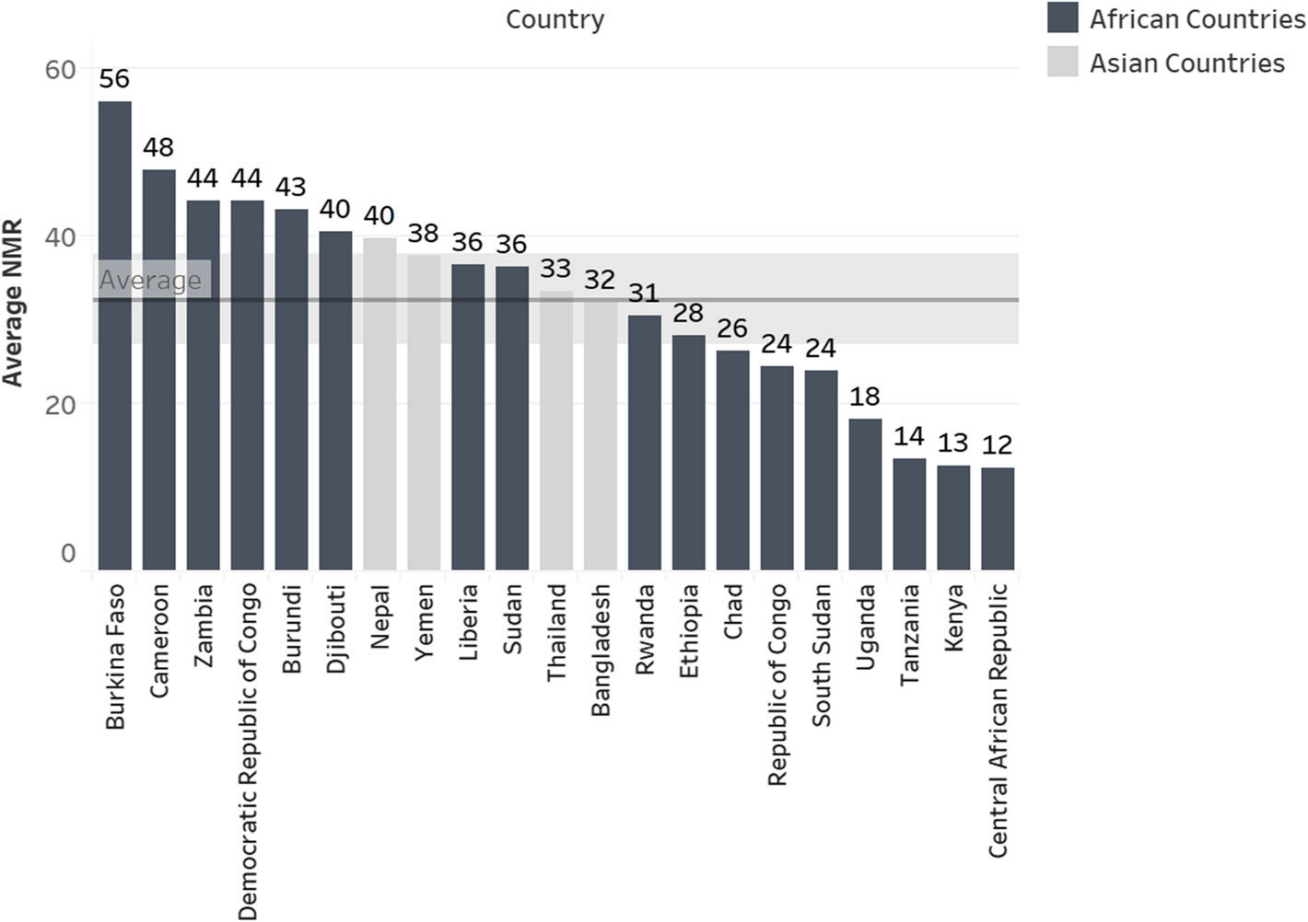
Average NMR for refugee camp populations by country, 2006–2017, based on reports from months with one or more neonatal deaths reported in the UNHCR HIS

Average annual and country-specific refugee camp NMRs are presented in Figs. 3 and 4, respectively. When refugee camp data from all countries using the UNHCR HIS are pooled, there appears to be little change in the average NMR over time. (Fig. 3) However, pooling data may mask disparities in NMRs across sites. Average NMRs for 2006–2017 ranged from a high of 56 neonatal deaths per 1000 live births in Burkina Faso refugee camps that reported data over a five-year period (2013–2017) to a low of 12 neonatal deaths per 1000 live births in Central African Republic camps that only reported data in 2013. (Fig. 4). Country-specific rates based on reports from months with one or more neonatal deaths reported in the UNHCR HIS are presented by year in Table 1.

**Table 1:**
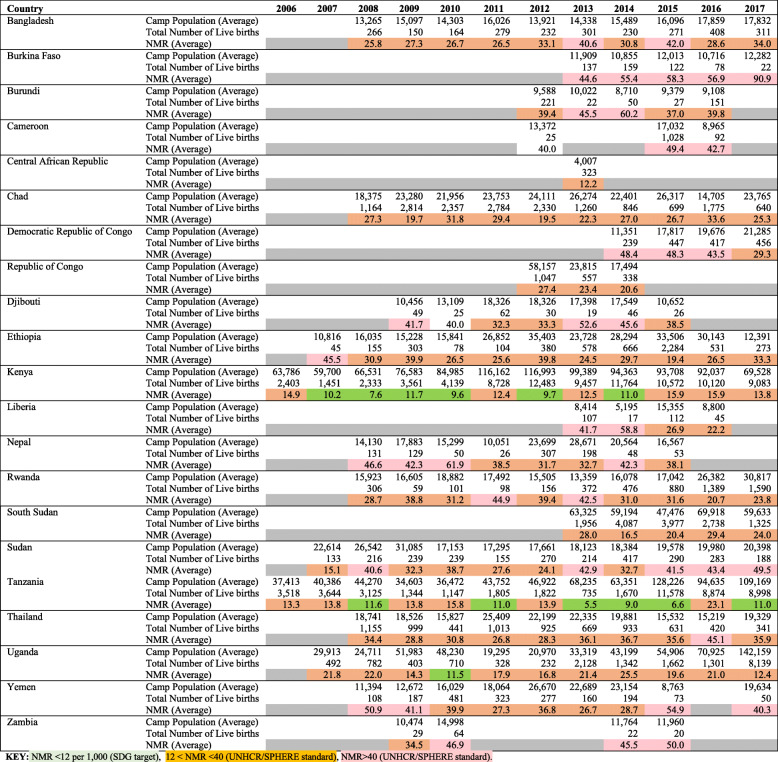
Refugee camp population, livebirths and neonatal mortality rate by country/year, based on months with one or more neonatal deaths reported in the UNHCR Health Information System

This analysis reveals a considerable number of countries (62%, *n* = 13) had annual refugee NMRs higher than the Sphere Standard, a well-established set of minimum standards for humanitarian response, of 40 per 1000 live births at some point during the study period. High NMR (above 40) was reported for at least three consecutive years in Burkina Faso, the Democratic Republic of Congo, Nepal and Sudan. Only three countries (Tanzania, Kenya and Uganda) had annual refugee camp NMRs less than or equal to the Sustainable Development Goal target of 12 newborn deaths per 1000 livebirths. Tanzania and Kenya both reported NMRs of 12 or less for six of the 12 years studied. Tanzania reported a NMR of 12 or less for 6 years with a minimum NMR of 6 in 2013 and maximum NMR of 16 in 2010. Similarly, Kenya reported NMR of 12 or less for 6 years with a minimum NMR of 8 in 2008 and a maximum NMR of 16 in 2016 and 2017, while Uganda reported a low NMR of 11 newborn deaths per 1000 livebirths only in 2010. Detailed rates for each camp, with sensitivity analysis including/ excluding months with zero newborn deaths reported, are presented by year in Supplementary File 2*.*

**Fig. 5:**
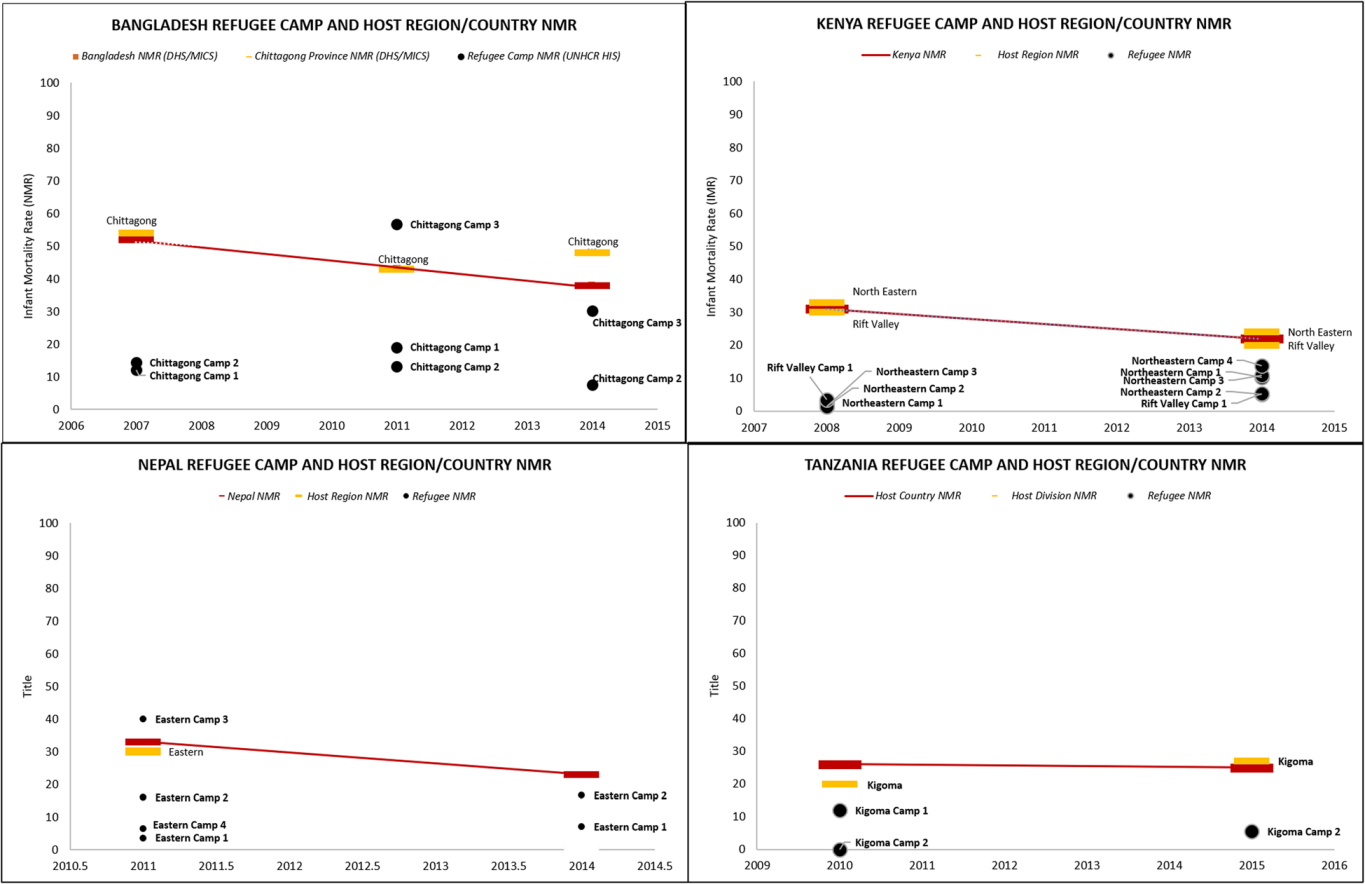
Select comparisons of refugee camp and host national neonatal mortality rates

In most countries and years where DHS or MICS data was available, refugee camp NMRs as reported in the UNHCR HIS was lower than that of the immediate host community (subnational survey division where camp is located) or national average. Figure 5 shows example of refugee camp NMRs calculated using UNHCR HIS data compared with host population NMR estimated from population-based survey data in selected African and Asian countries. Annex 3 (Supplementary Materials) presents NMRs by location and year for refugee camps and national/subnational host populations, where available; 60% of locations/years in this sub-analysis indicated that the neonatal mortality burden among refugees is likely lower than that of immediately surrounding host communities.

## Discussion

Understanding what has worked well and what needs to work better to ensure newborns survive and, ideally, thrive in humanitarian settings requires stakeholder commitment, reliable data, and sustained funding for both health service delivery. and implementation research. Although residents of refugee camps constitute a relatively small proportion of conflict-affected, forcibly displaced, marginalized and stateless populations that go uncounted in population-based surveys and vital registration systems, reporting and analyzing neonatal mortality burden and trends among this population is a critical step in strengthening data use and accountability for neonatal health in humanitarian settings. The UNHCR Health Information System provides insights into neonatal mortality burden and trends among refugee camp residents, as well as data quality issues to consider in design and use of routine health information systems to monitor neonatal health in sub-national populations.

Although NMRs in stable refugee camps are often lower than surrounding host populations, the neonatal mortality burden is still too high. Given the widespread recognition that perinatal outcomes are often misclassified and underreported, [23–25] and that there are still major gaps in stillbirth data in the UNHCR HIS, it is likely that neonatal mortality is underreported, and the true burden in refugee camp populations may be even higher than the upper bounds of figures presented here.

Even in countries hosting refugees in protracted camp situations, there has been limited change in the neonatal mortality burden over the last decade, although one would expect a decline over time. This may be due in part to limited attention to newborn health in humanitarian settings prior to the development of the *Newborn Health in Humanitarian Settings Field Guide* and UNHCR’s *Operational Guidelines on Improving Newborn Health in Refugee Operations* in 2014–2015 [9, 26]. The across and within country variation in NMR, and lack of change over time, suggests that concerted efforts are needed to further understand and address neonatal mortality. UNHCR has already begun this work in some settings, including the establishment of a neonatal mortality audit system in Jordan, and projects focused on improving access to quality health services for women and newborns in Cameroon, Chad, Jordan, Kenya, Niger and South Sudan [27–30]. Continued investment is needed in these and other refugee settings, including efforts to strengthen data availability and use as part of quality improvement efforts, as outlined in the recently launched *Roadmap to Accelerate Progress for Every Newborn in Humanitarian Settings 2020–2024* [31].

Our analysis has several limitations. First, incomplete and/or inaccurate reporting of routine data and outdated population size estimates can lead to implausibly high or low mortality estimates. In some cases, high average NMRs over the study period may be explained by low numbers of births reported in certain years. For example, low camp refugee population and live births numbers in Burkina Faso might explain NMRs reported in 2015–2017. Second, although UNHCR guidelines suggest that all deaths occurring within a camp should be reported in the HIS, stillbirths and neonatal deaths taking place outside of health facilities may be underreported. Third, the UNHCR HIS does not differentiate between early or late neonatal deaths, and neonatal death audits are not universal, both of which are critical for identifying and addressing gaps in accessibility and quality of care. Finally, population-based survey data for sub-national divisions where refugee camps are located also have limitations and cannot be directly compared to routine health information system data [32]. Surveys typically use a recall period of up to 2 years prior to survey administration; because availability of refugee camp HIS data varied from year to year, we compared NMRs reported in population-based surveys to NMRs reported in the UNHCR HIS in the year the survey was published, not the years where reported deaths occurred.

Data completeness and quality issues identified through this analysis point to opportunities for strengthening the UNHCR HIS to better facilitate monitoring and promote accountability for efforts to improve newborn health. These include increasing attention to the documentation of stillbirths in health facility registers and HIS reports; accurate differentiation of stillbirths and early neonatal deaths; strengthening community health worker engagement in identification and reporting of stillbirths and newborn deaths occurring outside of health facilities; establishing mechanisms to distinguish between missing data and reports of zero deaths in a given month; incorporating alerts to flag potentially implausible ratios among neonatal, infant and child mortality in HIS; and consistently reporting stillbirth and neonatal mortality rates alongside infant and child mortality in public health reports. World Health Organization guidance cautions against calculating case fatality rates for time periods when the number of deaths is too small for a stable calculation [33, 34]. It may be advisable for facilities with few deaths to calculate NMR on a quarterly or even annual basis for increased stability of the indicator. Future questions to be researched include at which levels of mortality is a stable rate produced, how frequently indicators should be calculated, and how to account for factors unique to refugee camp settings, including in/out migration, camp and health facility consolidation during time periods selected for calculation of NMR, which can exacerbate challenges in obtaining accurate denominators (numbers of live births) for mortality rate calculation. Learning from these efforts may also inform efforts to strengthen routine health information systems and maximize the use of routine data for monitoring and evaluation of health development efforts [35–37]. Outside of humanitarian settings, there are efforts to develop practical methods for improving measurements of perinatal mortality (stillbirth and early neonatal mortality) in health facilities [38], and strengthen maternal and perinatal death surveillance and response systems which may provide models for replication in refugee camps [39–41].

## Conclusions

The perinatal period represents a critical time of vulnerability and risk for newborns. Accurate and reliable data on mortality rates during this period is critical for improving access and quality of care for settings. The UNHCR HIS is one of the most comprehensive sources of information on refugee population health service coverage and outcomes. However, its functionality for tracking and analysis of neonatal mortality is quite limited. Concerted efforts and investments in strengthening both community surveillance and facility-based documentation are needed to ensure every neonatal death and stillbirth are captured in routine data collection, monitoring, and reporting, so that no populations are left behind in efforts to improve maternal and newborn health and well-being.

## Supplementary Information

**Additional file 1: Supplementary File 1.** Availability of neonatal mortality data in 120 refugee camps included in analysis.

**Additional file 2: Supplementary File 2.** Refugee camp population, livebirths and neonatal mortality rate reported in UNHCR HIS by country, camp and year, 2006–2017.

**Additional file 3: Supplementary File 3.** Neonatal mortality rates reported in UNHCR HIS and host population surveys by location and year, 2008–2016.

## Data Availability

All data analysed in this study are presented in the published article and its supplementary files. The full datasets are available from UNHCR upon reasonable request via https://his.unhcr.org/.

## Abbreviations

DHS: Demographic and Health Survey
HIS: Health Information System
MICS: Multiple Indicator Cluster Survey
NMR: Neonatal Mortality Rate
UNHCR: United Nations High Commissioner for Refugees
