# Supplementary material for: Neonatal mortality burden and trends in UNHCR refugee camps, 2006–2017: a retrospective analysis
Source: BMC Public Health. 2021 Feb 22;21:390. doi: 10.1186/s12889-021-10343-5 (PMC7898433; doi:10.1186/s12889-021-10343-5)
Supplement: Supplementary file 1 — Additional file 1: Supplementary File 1. Availability of neonatal mortality data in 120 refugee camps included in analysis. [file 12889_2021_10343_MOESM1_ESM.docx]

**Supplementary File 1: Availability of neonatal mortality data in 120 refugee camps included in analysis (months)**

| *Country* | Camp | 2006 | 2007 | 2008 | 2009 | 2010 | 2011 | 2012 | 2013 | 2014 | 2015 | 2016 | 2017 |
| --- | --- | --- | --- | --- | --- | --- | --- | --- | --- | --- | --- | --- | --- |
| *Bangladesh* | Kutupalong |  |  | 12 | 12 | 11 | 12 | 12 | 12 | 12 | 12 | 12 | 8 |
|  | Leda Site |  |  |  |  |  | 10 | 11 | 12 | 12 |  |  |  |
|  | Nayapara |  |  | 12 | 12 | 11 | 12 | 12 | 12 | 12 | 12 | 12 | 9 |
| *Burkina Faso* | Goudebo |  |  |  |  |  |  |  | 8 | 10 | 11 | 9 | 7 |
|  | Mentao |  |  |  |  |  |  |  | 10 | 9 | 11 | 6 | 6 |
| *Burundi* | Bwagiriza |  |  |  |  |  |  | 12 | 12 | 10 | 12 | 11 |  |
|  | Gasorwe |  |  |  |  |  |  | 11 | 12 | 11 | 11 | 12 |  |
|  | Kavumu |  |  |  |  |  |  |  |  | 9 | 12 | 12 |  |
|  | Musasa |  |  |  |  |  |  | 10 | 9 | 8 | 7 | 11 |  |
| *Cameroon* | Djohong |  |  |  |  |  |  |  | 11 |  |  |  |  |
|  | Kette |  |  |  |  |  |  | 6 |  |  |  |  |  |
|  | Mbile |  |  |  |  |  |  |  |  |  | 9 | 6 |  |
|  | Mborgop |  |  |  |  |  |  |  |  |  | 10 | 10 |  |
|  | Ndelele |  |  |  |  |  |  |  |  |  | 12 |  |  |
|  | Ngam |  |  |  |  |  |  |  |  |  |  | 11 |  |
| *Central African Republic* | Batalimo |  |  |  |  |  |  | 7 | 10 |  |  |  |  |
|  | Pladama |  |  |  |  |  |  |  | 10 |  |  |  |  |
| *Chad* | Amboko |  |  | 8 | 9 | 7 | 11 | 10 | 6 | 11 | 9 | 11 | 6 |
|  | Amnabak |  |  | 11 | 11 | 11 | 11 | 12 | 9 | 11 | 12 | 12 | 9 |
|  | Belom |  |  |  |  |  |  |  | 9 | 12 | 12 | 12 | 9 |
|  | Bredjing |  |  |  | 11 | 12 | 12 | 12 | 7 | 12 | 11 | 12 | 9 |
|  | Daha |  |  |  |  | 11 |  |  |  |  |  |  |  |
|  | Dar Es Salam |  |  |  |  |  |  |  |  |  |  | 10 | 8 |
|  | Djabal |  |  | 12 | 12 | 10 | 11 | 11 | 8 | 11 | 12 | 12 | 8 |
|  | Dosseye |  |  | 11 | 12 | 11 | 10 | 10 |  | 12 | 12 | 12 |  |
|  | Farchana |  |  |  | 12 | 12 | 12 | 11 | 12 | 12 | 12 | 12 | 9 |
|  | Gaga |  |  | 12 | 12 | 12 | 12 | 11 | 6 | 12 | 12 | 12 | 9 |
|  | Gassire |  |  |  |  |  | 6 |  |  |  |  |  |  |
|  | Gondje |  |  | 9 | 10 |  | 7 | 9 | 9 |  | 7 | 8 |  |
|  | Gouroukoune |  |  |  |  |  | 7 |  |  |  |  |  |  |
|  | Goz Amer |  |  | 12 | 12 | 10 | 11 | 10 | 9 | 11 | 12 | 12 | 9 |
|  | Iridimi |  |  |  |  | 7 | 11 | 11 | 12 | 12 | 12 | 12 | 9 |
|  | Koloma |  |  |  |  |  | 7 |  |  |  |  |  |  |
|  | Koubigou |  |  |  |  |  | 6 |  |  |  |  |  |  |
|  | Kounoungou |  |  | 12 | 12 | 12 | 9 | 11 | 11 | 12 | 12 | 12 | 9 |
|  | Mile |  |  | 12 | 12 | 11 | 9 | 10 | 10 | 12 | 12 | 12 | 8 |
|  | Moula |  |  |  | 11 | 7 | 9 | 7 |  |  |  |  |  |
|  | Moyo |  |  |  |  |  | 6 | 10 | 7 | 11 | 10 | 9 | 9 |
|  | Oure Cassoni |  |  | 12 | 12 | 12 | 12 | 11 | 10 | 11 | 12 | 12 | 8 |
|  | Touloum |  |  |  |  |  | 12 | 12 | 12 | 12 | 12 | 12 | 9 |
|  | Treguine |  |  | 12 | 11 | 12 | 12 | 12 | 7 | 12 | 11 | 12 | 6 |
|  | Yaroungou |  |  | 6 |  | 11 | 8 | 10 |  |  |  |  |  |
| *Democratic Republic of Congo* | Bili |  |  |  |  |  |  |  |  |  |  | 12 | 8 |
|  | Boyabo |  |  |  |  |  |  |  |  | 12 | 11 | 12 | 8 |
|  | Inke |  |  |  |  |  |  |  |  | 9 | 12 | 9 | 8 |
|  | Lusenda |  |  |  |  |  |  |  |  |  |  | 12 | 8 |
|  | Mole |  |  |  |  |  |  |  |  | 10 | 12 | 12 | 8 |
| *Republic of Congo* | Betou |  |  |  |  |  |  |  | 12 | 6 |  |  |  |
|  | Impfondo |  |  |  |  |  |  | 11 | 8 |  |  |  |  |
| *Djibouti* | Ali Adde |  |  | 7 | 10 | 11 | 10 | 12 | 11 | 11 | 10 | 9 | 6 |
| *Ethiopia* | Awbarre |  |  | 8 | 9 | 11 | 11 | 9 | 11 | 12 | 12 | 12 | 6 |
|  | Aysaita |  |  |  |  |  |  |  |  | 7 |  |  |  |
|  | Bambasi |  |  |  |  |  |  |  | 9 | 12 | 12 | 10 | 8 |
|  | Barahle |  |  |  |  |  |  |  |  |  |  | 8 |  |
|  | Bokolmanyo |  |  |  |  | 11 | 12 | 12 | 11 | 12 | 12 | 11 | 7 |
|  | Bonga |  | 11 |  |  |  |  |  |  |  |  |  |  |
|  | Buramino |  |  |  |  |  |  |  | 12 | 12 | 12 | 10 | 8 |
|  | Dimma |  | 6 |  |  |  |  |  |  |  |  |  |  |
|  | Fugnido |  | 12 | 12 | 12 | 12 | 6 | 10 | 12 | 12 | 12 | 12 | 6 |
|  | Fugnido 2 |  |  |  |  |  |  |  |  |  |  | 8 | 8 |
|  | Hilaweyn |  |  |  |  |  |  |  | 9 | 10 | 11 | 9 | 8 |
|  | Jewi |  |  |  |  |  |  |  |  |  |  | 11 | 9 |
|  | Kebribeyah |  | 12 | 11 | 11 | 12 | 11 | 9 | 12 | 12 | 12 | 12 | 8 |
|  | Kobe |  |  |  |  |  |  | 6 | 12 | 12 | 12 | 10 | 7 |
|  | Kule |  |  |  |  |  |  |  |  | 7 | 10 | 12 | 8 |
|  | Leitchuor |  |  |  |  |  |  |  |  | 7 | 6 |  |  |
|  | Mai Aini |  |  |  |  |  | 11 | 10 | 12 | 10 |  | 8 |  |
|  | Melkadida |  |  |  |  |  | 12 | 11 | 12 | 12 | 11 | 10 | 8 |
|  | Nguenyyiel |  |  |  |  |  |  |  |  |  |  |  | 6 |
|  | Okugo |  |  |  |  |  |  |  |  |  | 10 | 9 | 7 |
|  | Sheder |  |  |  |  | 8 | 11 | 11 | 10 | 8 | 12 | 12 | 8 |
|  | Sherkole |  | 11 |  |  |  |  | 11 |  | 11 | 12 | 12 | 8 |
|  | Shimelba |  | 11 | 11 | 9 | 9 | 7 | 6 |  |  |  |  |  |
|  | Tierkidi |  |  |  |  |  |  |  |  | 8 | 11 | 10 | 8 |
|  | Tongo |  |  |  |  |  |  | 9 | 9 | 11 | 12 | 12 | 7 |
|  | Tsore |  |  |  |  |  |  |  |  |  |  |  | 8 |
| *Kenya* | Dagahaley | 10 | 11 | 12 | 12 | 12 | 12 | 12 | 12 | 12 | 12 | 12 | 10 |
|  | Hagadera | 11 | 11 | 12 | 12 | 12 | 12 | 12 | 12 | 12 | 12 | 12 | 10 |
|  | Ifo | 10 | 11 | 12 | 12 | 12 | 12 | 12 | 12 | 12 | 12 | 12 | 10 |
|  | Ifo 2 |  |  |  |  |  |  | 8 | 12 | 12 | 12 | 12 | 10 |
|  | Kakuma | 12 | 12 | 12 | 12 | 12 | 12 | 12 | 12 | 12 | 12 | 12 | 10 |
|  | Kalobeyei |  |  |  |  |  |  |  |  |  |  |  | 8 |
|  | Kambioos |  |  |  |  |  |  | 10 | 12 | 12 | 12 | 12 |  |
| *Liberia* | Bahn |  |  |  |  |  |  |  | 7 | 8 |  |  |  |
|  | PTP |  |  |  |  |  |  |  | 9 | 7 | 11 | 11 |  |
| *Nepal* | Beldangi |  |  |  |  |  |  | 12 | 12 | 12 | 10 |  |  |
|  | Beldangi I |  |  | 10 | 11 | 10 |  |  |  |  |  |  |  |
|  | Beldangi II |  |  | 11 | 12 | 9 |  |  |  |  |  |  |  |
|  | Beldangi II ext |  |  | 7 | 7 |  |  |  |  |  |  |  |  |
|  | Khudunabari |  |  | 7 | 8 | 9 |  |  |  |  |  |  |  |
|  | Sanishare |  |  | 10 | 11 | 10 | 9 | 7 |  |  |  |  |  |
|  | Timai |  |  | 7 | 9 |  |  |  |  |  |  |  |  |
| *Rwanda* | Gihembe |  |  | 8 | 6 | 7 | 10 | 12 | 12 | 12 | 12 | 12 | 9 |
|  | Kigeme |  |  |  |  |  |  |  |  | 12 | 12 | 12 | 9 |
|  | Kiziba |  |  | 8 | 6 | 12 | 12 | 12 | 12 | 12 | 12 | 12 | 9 |
|  | Mahama |  |  |  |  |  |  |  |  |  | 8 | 12 | 9 |
|  | Mugombwa |  |  |  |  |  |  |  |  |  | 9 | 11 | 9 |
|  | Nyabiheke |  |  | 6 | 7 | 8 | 11 | 10 | 12 | 12 | 12 | 12 | 9 |
| *South Sudan* | Adjoung Thok |  |  |  |  |  |  |  |  |  |  | 12 | 8 |
|  | Bunj Hospital |  |  |  |  |  |  |  |  |  |  | 8 | 6 |
|  | Doro |  |  |  |  |  |  |  | 6 |  | 12 | 12 | 7 |
|  | Gendrassa |  |  |  |  |  |  |  |  | 9 | 12 | 10 | 8 |
|  | Gentil Hospital |  |  |  |  |  |  |  |  |  |  |  | 7 |
|  | Gorom |  |  |  |  |  |  |  |  | 12 |  |  |  |
|  | Kaya |  |  |  |  |  |  |  |  |  | 12 | 12 | 7 |
|  | Lasu |  |  |  |  |  |  |  | 10 | 11 | 12 | 6 |  |
|  | Pamir |  |  |  |  |  |  |  |  |  |  |  | 8 |
|  | Panrieng |  |  |  |  |  |  |  |  |  |  |  | 8 |
|  | Yida |  |  |  |  |  |  |  | 11 | 12 | 12 | 12 | 8 |
|  | Yusuf Batil |  |  |  |  |  |  |  |  | 11 | 6 | 12 | 6 |
| *Sudan* | Fau 5 |  |  |  |  |  |  |  | 6 |  |  |  |  |
|  | Girba |  |  | 7 |  |  | 12 | 8 | 10 | 12 | 12 | 12 | 9 |
|  | Kilo 26 |  |  | 11 | 11 | 10 | 12 | 7 | 11 | 11 | 12 | 11 | 9 |
|  | Shagarab I II III |  | 6 | 12 | 12 | 11 | 12 | 8 | 12 | 12 | 12 | 12 | 9 |
|  | Um Gargour |  | 6 | 12 | 11 | 9 | 11 |  | 10 | 12 | 9 | 12 | 9 |
|  | Wad Sharifey |  |  | 10 | 10 | 10 | 12 | 8 | 12 | 12 | 12 | 12 | 9 |
| *Tanzania* | Kanembwa | 12 | 11 |  |  |  |  |  |  |  |  |  |  |
|  | Lugufu | 12 | 12 | 12 | 8 |  |  |  |  |  |  |  |  |
|  | Lugufu II | 12 | 8 |  |  |  |  |  |  |  |  |  |  |
|  | Lukole | 12 | 12 | 6 |  |  |  |  |  |  |  |  |  |
|  | Mtabila | 12 | 12 | 12 | 12 | 12 | 12 | 11 |  |  |  |  |  |
|  | Mtabila II | 12 | 12 |  |  |  |  |  |  |  |  |  |  |
|  | Mtendeli | 12 |  |  |  |  |  |  |  |  |  | 6 | 9 |
|  | Muyovosi | 12 |  |  |  |  |  |  |  |  |  |  |  |
|  | Nduta | 12 | 12 | 11 |  |  |  |  |  |  |  | 12 | 7 |
|  | Nyarugusu | 12 | 12 | 12 | 12 | 12 | 12 | 12 | 11 | 12 | 12 | 11 | 8 |
| *Thailand* | Ban Mai Nai Soi |  |  | 12 | 12 | 12 | 12 | 12 | 12 | 10 | 10 | 7 |  |
|  | Mae La |  |  | 12 | 12 | 12 | 12 | 12 | 12 | 12 | 12 | 12 | 9 |
|  | Mae La Oon |  |  | 12 | 12 | 12 | 12 | 12 | 12 | 12 | 11 | 12 | 9 |
|  | Mae Ra Ma Luang |  |  | 12 | 12 | 12 | 12 | 12 | 12 | 12 | 11 | 12 | 9 |
|  | Nu Poh |  |  | 12 | 12 | 12 | 12 | 12 | 12 | 12 | 12 | 10 | 8 |
|  | Tham Hin |  |  | 11 | 10 | 10 | 10 | 10 | 10 | 9 | 9 |  |  |
|  | Umpiem Mai |  |  | 12 | 12 | 12 | 12 | 12 | 12 | 12 | 12 | 12 | 8 |
| *Uganda* | Adjumani |  | 7 | 12 | 6 |  |  |  |  | 10 | 12 | 12 | 9 |
|  | Bidibidi |  |  |  |  |  |  |  |  |  |  |  | 9 |
|  | Imvepi |  |  |  |  |  |  |  |  |  |  |  | 6 |
|  | Kiryandongo |  | 6 |  |  |  |  |  |  |  | 12 | 12 | 8 |
|  | Kyaka II |  | 7 | 11 | 12 | 12 | 12 | 12 | 12 | 12 | 12 | 12 | 9 |
|  | Kyangwali |  | 6 | 12 | 12 | 12 | 12 | 12 | 12 | 12 | 12 | 12 | 9 |
|  | Nakivale |  | 7 | 12 | 12 | 12 | 12 | 11 | 12 | 12 | 12 | 12 | 9 |
|  | Nyakabande |  |  |  |  |  |  | 9 |  |  |  |  |  |
|  | Oruchinga |  |  |  |  |  |  | 10 |  |  |  | 10 |  |
|  | Palorinya |  | 6 | 11 |  |  |  |  |  |  |  |  | 9 |
|  | Rhino Camp |  |  |  |  |  |  |  |  | 10 | 11 | 11 | 9 |
|  | Rwamwanja |  |  |  |  |  |  | 7 | 12 | 12 | 12 | 12 | 9 |
| *Yemen* | Basateen |  |  |  | 7 | 12 | 12 | 11 | 12 | 10 |  |  | 7 |
|  | Kharaz |  |  | 7 | 9 | 12 | 12 | 11 | 11 | 12 | 11 | 10 | 8 |
|  | Sanaa |  |  |  | 9 | 12 | 12 | 11 | 10 |  | 8 | 9 |  |
| *Zambia* | Maheba |  |  |  | 10 | 9 | 12 |  |  | 11 | 12 | 11 | 7 |
|  | Mayukwayukwa |  |  | 6 | 10 |  | 12 |  |  | 11 | 12 | 7 |  |
