# Supplementary material for: Neonatal mortality burden and trends in UNHCR refugee camps, 2006–2017: a retrospective analysis
Source: BMC Public Health. 2021 Feb 22;21:390. doi: 10.1186/s12889-021-10343-5 (PMC7898433; doi:10.1186/s12889-021-10343-5)
Supplement: Supplementary file 2 — Additional file 2: Supplementary File 2. Refugee camp population, livebirths and neonatal mortality rate reported in UNHCR HIS by country, camp and year, 2006–2017. [file 12889_2021_10343_MOESM2_ESM.docx]

**Annex 2: Refugee camp population, livebirths and neonatal mortality rate reported in UNHCR HIS by country, camp and year, 2006-2017**

| **Country** | **Camp** |  | **2006** | **2007** | **2008** | **2009** | **2010** | **2011** | **2012** | **2013** | **2014** | **2015** | **2016** | **2017** |
| --- | --- | --- | --- | --- | --- | --- | --- | --- | --- | --- | --- | --- | --- | --- |
| **Bangladesh** | **Kutupalong** | Average camp population |  |  | 10,932 | 11,133 | 11,361 | 11,612 | 12,191 | 12,516 | 12,947 | 13,200 | 13,688 | 13,912 |
|  |  | Total Number of live births |  |  | 505 | 495 | 345 | 327 | 463 | 397 | 373 | 460 | 428 | 302 |
|  |  | # of months (including zero entries) |  |  | 12 | 12 | 11 | 12 | 12 | 12 | 12 | 12 | 12 | 8 |
|  |  | Average NMR (Zero-included) |  |  | 8 | 2 | 6 | 0 | 2 | 6 | 0 | 11 | 4 | 11 |
|  |  | # of months (excluding zero entries) |  |  | 3 | 1 | 2 |  | 1 | 3 |  | 3 | 2 | 2 |
|  |  | Average NMR (zero-excluded) |  |  | 33 | 28 | 34 |  | 23 | 25 |  | 46 | 21 | 42 |
|  | **Leda Site** | Average camp population |  |  |  |  |  | 13,834 | 14,531 | 14,645 | 14,976 |  |  |  |
|  |  | Total Number of live births |  |  |  |  |  | 427 | 457 | 417 | 369 |  |  |  |
|  |  | # of months (including zero entries) |  |  |  |  |  | 10 | 11 | 12 | 12 |  |  |  |
|  |  | Average NMR (Zero-included) |  |  |  |  |  | 8 | 13 | 16 | 15 |  |  |  |
|  |  | # of months (excluding zero entries) |  |  |  |  |  | 3 | 4 | 4 | 6 |  |  |  |
|  |  | Average NMR (zero-excluded) |  |  |  |  |  | 25 | 36 | 47 | 30 |  |  |  |
|  | **Nayapara** | Average camp population |  |  | 16,941 | 17,091 | 17,367 | 17,634 | 17,983 | 18,367 | 18,803 | 19,123 | 19,213 | 19,401 |
|  |  | Total Number of live births |  |  | 772 | 605 | 510 | 474 | 606 | 562 | 566 | 617 | 571 | 420 |
|  |  | # of months (including zero entries) |  |  | 12 | 12 | 11 | 12 | 12 | 12 | 12 | 12 | 12 | 9 |
|  |  | Average NMR (Zero-included) |  |  | 2 | 5 | 4 | 9 | 0 | 5 | 3 | 10 | 15 | 17 |
|  |  | # of months (excluding zero entries) |  |  | 2 | 2 | 2 | 4 |  | 1 | 1 | 3 | 6 | 5 |
|  |  | Average NMR (zero-excluded) |  |  | 15 | 27 | 20 | 27 |  | 61 | 34 | 38 | 31 | 31 |
| **Burkina Faso** | **Goudebo** | Average camp population |  |  |  |  |  |  |  | 10,363 | 9,409 | 10,161 | 9,886 | 9,570 |
|  |  | Total Number of live births |  |  |  |  |  |  |  | 215 | 257 | 286 | 213 | 158 |
|  |  | # of months (including zero entries) |  |  |  |  |  |  |  | 8 | 10 | 11 | 9 | 7 |
|  |  | Average NMR (Zero-included) |  |  |  |  |  |  |  | 19 | 12 | 3 | 13 | 0 |
|  |  | # of months (excluding zero entries) |  |  |  |  |  |  |  | 3 | 2 | 1 | 2 |  |
|  |  | Average NMR (zero-excluded) |  |  |  |  |  |  |  | 52 | 61 | 37 | 59 |  |
|  | **Mentao** | Average camp population |  |  |  |  |  |  |  | 16,568 | 11,748 | 12,755 | 12,553 | 12,372 |
|  |  | Total Number of live births |  |  |  |  |  |  |  | 265 | 206 | 219 | 116 | 122 |
|  |  | # of months (including zero entries) |  |  |  |  |  |  |  | 10 | 9 | 11 | 6 | 6 |
|  |  | Average NMR (Zero-included) |  |  |  |  |  |  |  | 2 | 23 | 28 | 9 | 15 |
|  |  | # of months (excluding zero entries) |  |  |  |  |  |  |  | 1 | 4 | 5 | 1 | 1 |
|  |  | Average NMR (zero-excluded) |  |  |  |  |  |  |  | 24 | 52 | 62 | 53 | 91 |
| **Burundi** | **Bwagiriza** | Average camp population |  |  |  |  |  |  | 8,088 | 10,133 | 9,798 | 9,541 | 8,932 |  |
|  |  | Total Number of live births |  |  |  |  |  |  | 299 | 404 | 212 | 281 | 325 |  |
|  |  | # of months (including zero entries) |  |  |  |  |  |  | 12 | 12 | 10 | 12 | 11 |  |
|  |  | Average NMR (Zero-included) |  |  |  |  |  |  | 6 | 0 | 12 | 3 | 14 |  |
|  |  | # of months (excluding zero entries) |  |  |  |  |  |  | 2 |  | 2 | 1 | 4 |  |
|  |  | Average NMR (zero-excluded) |  |  |  |  |  |  | 33 |  | 59 | 37 | 37 |  |
|  | **Gasorwe** | Average camp population |  |  |  |  |  |  | 9,844 | 9,929 | 9,831 | 9,781 | 8,785 |  |
|  |  | Total Number of live births |  |  |  |  |  |  | 275 | 321 | 279 | 263 | 312 |  |
|  |  | # of months (including zero entries) |  |  |  |  |  |  | 11 | 12 | 11 | 11 | 12 |  |
|  |  | Average NMR (Zero-included) |  |  |  |  |  |  | 23 | 4 | 0 | 0 | 0 |  |
|  |  | # of months (excluding zero entries) |  |  |  |  |  |  | 6 | 1 |  |  |  |  |
|  |  | Average NMR (zero-excluded) |  |  |  |  |  |  | 41 | 45 |  |  |  |  |
|  | **Kavumu** | Average camp population |  |  |  |  |  |  |  |  | 5,608 | 7,689 | 10,360 |  |
|  |  | Total Number of live births |  |  |  |  |  |  |  |  | 188 | 278 | 341 |  |
|  |  | # of months (including zero entries) |  |  |  |  |  |  |  |  | 9 | 12 | 12 |  |
|  |  | Average NMR (Zero-included) |  |  |  |  |  |  |  |  | 7 | 0 | 4 |  |
|  |  | # of months (excluding zero entries) |  |  |  |  |  |  |  |  | 1 |  | 1 |  |
|  |  | Average NMR (zero-excluded) |  |  |  |  |  |  |  |  | 63 |  | 50 |  |
|  | **Musasa** | Average camp population |  |  |  |  |  |  | 6,482 | 6,902 | 6,911 | 7,034 | 6,668 |  |
|  |  | Total Number of live births |  |  |  |  |  |  | 179 | 173 | 167 | 142 | 217 |  |
|  |  | # of months (including zero entries) |  |  |  |  |  |  | 10 | 9 | 8 | 7 | 11 |  |
|  |  | Average NMR (Zero-included) |  |  |  |  |  |  | 0 | 0 | 0 | 0 | 0 |  |
|  |  | # of months (excluding zero entries) |  |  |  |  |  |  |  |  |  |  |  |  |
|  |  | Average NMR (zero-excluded) |  |  |  |  |  |  |  |  |  |  |  |  |
| **Cameroon** | **Djohong** | Average camp population |  |  |  |  |  |  |  | 20,217 |  |  |  |  |
|  |  | Total Number of live births |  |  |  |  |  |  |  | 481 |  |  |  |  |
|  |  | # of months (including zero entries) |  |  |  |  |  |  |  | 11 |  |  |  |  |
|  |  | Average NMR (Zero-included) |  |  |  |  |  |  |  | 6 |  |  |  |  |
|  |  | # of months (excluding zero entries) |  |  |  |  |  |  |  |  |  |  |  |  |
|  |  | Average NMR (zero-excluded) |  |  |  |  |  |  |  |  |  |  |  |  |
|  | **Kette** | Average camp population |  |  |  |  |  |  | 13,372 |  |  |  |  |  |
|  |  | Total Number of live births |  |  |  |  |  |  | 119 |  |  |  |  |  |
|  |  | # of months (including zero entries) |  |  |  |  |  |  | 6 |  |  |  |  |  |
|  |  | Average NMR (Zero-included) |  |  |  |  |  |  | 7 |  |  |  |  |  |
|  |  | # of months (excluding zero entries) |  |  |  |  |  |  | 1 |  |  |  |  |  |
|  |  | Average NMR (zero-excluded) |  |  |  |  |  |  | 40 |  |  |  |  |  |
|  | **Mbile** | Average camp population |  |  |  |  |  |  |  |  |  | 10,730 | 11,437 |  |
|  |  | Total Number of live births |  |  |  |  |  |  |  |  |  | 342 | 205 |  |
|  |  | # of months (including zero entries) |  |  |  |  |  |  |  |  |  | 9 | 6 |  |
|  |  | Average NMR (Zero-included) |  |  |  |  |  |  |  |  |  | 48 | 9 |  |
|  |  | # of months (excluding zero entries) |  |  |  |  |  |  |  |  |  | 6 | 1 |  |
|  |  | Average NMR (zero-excluded) |  |  |  |  |  |  |  |  |  | 71 | 51 |  |
|  | **Mborgop** | Average camp population |  |  |  |  |  |  |  |  |  | 11,428 | 12,042 |  |
|  |  | Total Number of live births |  |  |  |  |  |  |  |  |  | 327 | 419 |  |
|  |  | # of months (including zero entries) |  |  |  |  |  |  |  |  |  | 10 | 10 |  |
|  |  | Average NMR (Zero-included) |  |  |  |  |  |  |  |  |  | 0 | 3 |  |
|  |  | # of months (excluding zero entries) |  |  |  |  |  |  |  |  |  |  | 1 |  |
|  |  | Average NMR (zero-excluded) |  |  |  |  |  |  |  |  |  |  | 33 |  |
|  | **Ndelele** | Average camp population |  |  |  |  |  |  |  |  |  | 20,463 |  |  |
|  |  | Total Number of live births |  |  |  |  |  |  |  |  |  | 850 |  |  |
|  |  | # of months (including zero entries) |  |  |  |  |  |  |  |  |  | 12 |  |  |
|  |  | Average NMR (Zero-included) |  |  |  |  |  |  |  |  |  | 34 |  |  |
|  |  | # of months (excluding zero entries) |  |  |  |  |  |  |  |  |  | 11 |  |  |
|  |  | Average NMR (zero-excluded) |  |  |  |  |  |  |  |  |  | 37 |  |  |
|  | **Ngam** | Average camp population |  |  |  |  |  |  |  |  |  |  | 5,490 |  |
|  |  | Total Number of live births |  |  |  |  |  |  |  |  |  |  | 298 |  |
|  |  | # of months (including zero entries) |  |  |  |  |  |  |  |  |  |  | 11 |  |
|  |  | Average NMR (Zero-included) |  |  |  |  |  |  |  |  |  |  | 4 |  |
|  |  | # of months (excluding zero entries) |  |  |  |  |  |  |  |  |  |  | 1 |  |
|  |  | Average NMR (zero-excluded) |  |  |  |  |  |  |  |  |  |  | 43 |  |
| **Central African Republic** | **Batalimo** | Average camp population |  |  |  |  |  |  | 6,997 | 5,987 |  |  |  |  |
|  |  | Total Number of live births |  |  |  |  |  |  | 1,169 | 2,750 |  |  |  |  |
|  |  | # of months (including zero entries) |  |  |  |  |  |  | 7 | 10 |  |  |  |  |
|  |  | Average NMR (Zero-included) |  |  |  |  |  |  | 0 | 1 |  |  |  |  |
|  |  | # of months (excluding zero entries) |  |  |  |  |  |  |  | 1 |  |  |  |  |
|  |  | Average NMR (zero-excluded) |  |  |  |  |  |  |  | 4 |  |  |  |  |
|  | **Pladama** | Average camp population |  |  |  |  |  |  |  | 2,027 |  |  |  |  |
|  |  | Total Number of live births |  |  |  |  |  |  |  | 489 |  |  |  |  |
|  |  | # of months (including zero entries) |  |  |  |  |  |  |  | 10 |  |  |  |  |
|  |  | Average NMR (Zero-included) |  |  |  |  |  |  |  | 2 |  |  |  |  |
|  |  | # of months (excluding zero entries) |  |  |  |  |  |  |  | 1 |  |  |  |  |
|  |  | Average NMR (zero-excluded) |  |  |  |  |  |  |  | 21 |  |  |  |  |
| **Chad** | **Amboko** | Average camp population |  |  | 11,997 | 11,973 | 11,072 | 11,159 | 11,508 | 11,699 | 10,827 | 11,576 | 8,284 | 8,645 |
|  |  | Total Number of live births |  |  | 178 | 178 | 142 | 235 | 190 | 111 | 214 | 218 | 219 | 114 |
|  |  | # of months (including zero entries) |  |  | 8 | 9 | 7 | 11 | 10 | 6 | 11 | 9 | 11 | 6 |
|  |  | Average NMR (Zero-included) |  |  | 4 | 0 | 0 | 0 | 0 | 0 | 0 | 0 | 0 | 7 |
|  |  | # of months (excluding zero entries) |  |  | 1 |  |  |  |  |  |  |  |  |  |
|  |  | Average NMR (zero-excluded) |  |  | 33 |  |  |  |  |  |  |  |  |  |
|  | **Amnabak** | Average camp population |  |  | 16,700 | 16,753 | 17,256 | 18,892 | 18,926 | 22,175 | 24,056 | 22,621 | 20,910 | 21,885 |
|  |  | Total Number of live births |  |  | 394 | 514 | 531 | 551 | 647 | 518 | 656 | 671 | 678 | 394 |
|  |  | # of months (including zero entries) |  |  | 11 | 11 | 11 | 11 | 12 | 9 | 11 | 12 | 12 | 9 |
|  |  | Average NMR (Zero-included) |  |  | 0 | 2 | 20 | 21 | 2 | 0 | 0 | 0 | 0 | 7 |
|  |  | # of months (excluding zero entries) |  |  |  | 1 | 5 | 3 | 1 |  |  |  |  | 2 |
|  |  | Average NMR (zero-excluded) |  |  |  | 19 | 44 | 41 | 19 |  |  |  |  | 30 |
|  | **Belom** | Average camp population |  |  |  |  |  |  |  | 21,641 | 23,796 | 23,713 | 18,768 | 19,715 |
|  |  | Total Number of live births |  |  |  |  |  |  |  | 463 | 499 | 645 | 613 | 384 |
|  |  | # of months (including zero entries) |  |  |  |  |  |  |  | 9 | 12 | 12 | 12 | 9 |
|  |  | Average NMR (Zero-included) |  |  |  |  |  |  |  | 10 | 19 | 4 | 18 | 16 |
|  |  | # of months (excluding zero entries) |  |  |  |  |  |  |  | 4 | 7 | 2 | 6 | 4 |
|  |  | Average NMR (zero-excluded) |  |  |  |  |  |  |  | 23 | 32 | 22 | 35 | 36 |
|  | **Bredjing** | Average camp population |  |  |  | 32,119 | 33,042 | 34,914 | 36,852 | 38,693 | 40,264 | 40,404 | 41,477 | 43,475 |
|  |  | Total Number of live births |  |  |  | 1,341 | 1,576 | 1,691 | 1,563 | 845 | 1,405 | 1,277 | 1,410 | 925 |
|  |  | # of months (including zero entries) |  |  |  | 11 | 12 | 12 | 12 | 7 | 12 | 11 | 12 | 9 |
|  |  | Average NMR (Zero-included) |  |  |  | 7 | 8 | 4 | 1 | 2 | 0 | 2 | 1 | 1 |
|  |  | # of months (excluding zero entries) |  |  |  | 6 | 7 | 4 | 2 | 2 |  | 2 | 1 | 1 |
|  |  | Average NMR (zero-excluded) |  |  |  | 13 | 13 | 12 | 7 | 7 |  | 8 | 9 | 9 |
|  | **Daha** | Average camp population |  |  |  |  | 11,485 |  |  |  |  |  |  |  |
|  |  | Total Number of live births |  |  |  |  | 367 |  |  |  |  |  |  |  |
|  |  | # of months (including zero entries) |  |  |  |  | 11 |  |  |  |  |  |  |  |
|  |  | Average NMR (Zero-included) |  |  |  |  | 0 |  |  |  |  |  |  |  |
|  |  | # of months (excluding zero entries) |  |  |  |  |  |  |  |  |  |  |  |  |
|  |  | Average NMR (zero-excluded) |  |  |  |  |  |  |  |  |  |  |  |  |
|  | **Dar Es Salam** | Average camp population |  |  |  |  |  |  |  |  |  |  | 4,947 | 5,879 |
|  |  | Total Number of live births |  |  |  |  |  |  |  |  |  |  | 318 | 1,703 |
|  |  | # of months (including zero entries) |  |  |  |  |  |  |  |  |  |  | 10 | 8 |
|  |  | Average NMR (Zero-included) |  |  |  |  |  |  |  |  |  |  | 17 | 0 |
|  |  | # of months (excluding zero entries) |  |  |  |  |  |  |  |  |  |  | 5 |  |
|  |  | Average NMR (zero-excluded) |  |  |  |  |  |  |  |  |  |  | 34 |  |
|  | **Djabal** | Average camp population |  |  | 16,120 | 17,235 | 16,668 | 17,469 | 18,534 | 18,968 | 19,911 | 20,978 | 20,540 | 21,308 |
|  |  | Total Number of live births |  |  | 653 | 712 | 630 | 820 | 672 | 460 | 719 | 707 | 696 | 502 |
|  |  | # of months (including zero entries) |  |  | 12 | 12 | 10 | 11 | 11 | 8 | 11 | 12 | 12 | 8 |
|  |  | Average NMR (Zero-included) |  |  | 5 | 4 | 0 | 0 | 1 | 0 | 0 | 0 | 0 | 2 |
|  |  | # of months (excluding zero entries) |  |  | 3 | 2 |  |  | 1 |  |  |  |  | 1 |
|  |  | Average NMR (zero-excluded) |  |  | 22 | 26 |  |  | 16 |  |  |  |  | 18 |
|  | **Dosseye** | Average camp population |  |  | 7,565 | 8,655 | 9,616 | 9,569 | 9,888 |  | 16,555 | 19,764 | 12,260 |  |
|  |  | Total Number of live births |  |  | 304 | 326 | 286 | 206 | 225 |  | 413 | 390 | 350 |  |
|  |  | # of months (including zero entries) |  |  | 11 | 12 | 11 | 10 | 10 |  | 12 | 12 | 12 |  |
|  |  | Average NMR (Zero-included) |  |  | 13 | 3 | 4 | 4 | 0 |  | 4 | 3 | 26 |  |
|  |  | # of months (excluding zero entries) |  |  | 3 | 1 | 1 | 1 |  |  | 2 | 1 | 7 |  |
|  |  | Average NMR (zero-excluded) |  |  | 47 | 38 | 43 | 36 |  |  | 24 | 31 | 44 |  |
|  | **Farchana** | Average camp population |  |  |  | 20,386 | 21,800 | 22,576 | 23,421 | 25,766 | 26,858 | 26,403 | 26,968 | 27,732 |
|  |  | Total Number of live births |  |  |  | 840 | 908 | 893 | 848 | 863 | 738 | 712 | 729 | 513 |
|  |  | # of months (including zero entries) |  |  |  | 12 | 12 | 12 | 11 | 12 | 12 | 12 | 12 | 9 |
|  |  | Average NMR (Zero-included) |  |  |  | 3 | 0 | 9 | 0 | 0 | 0 | 0 | 0 | 0 |
|  |  | # of months (excluding zero entries) |  |  |  | 1 |  | 3 |  |  |  |  |  |  |
|  |  | Average NMR (zero-excluded) |  |  |  | 37 |  | 37 |  |  |  |  |  |  |
|  | **Gaga** | Average camp population |  |  | 18,994 | 18,684 | 18,552 | 20,845 | 22,105 | 22,747 | 23,946 | 23,562 | 23,402 | 24,178 |
|  |  | Total Number of live births |  |  | 899 | 1,033 | 860 | 855 | 816 | 389 | 788 | 693 | 795 | 547 |
|  |  | # of months (including zero entries) |  |  | 12 | 12 | 12 | 12 | 11 | 6 | 12 | 12 | 12 | 9 |
|  |  | Average NMR (Zero-included) |  |  | 7 | 4 | 0 | 0 | 0 | 0 | 0 | 0 | 0 | 0 |
|  |  | # of months (excluding zero entries) |  |  | 4 | 3 |  |  |  |  |  |  |  |  |
|  |  | Average NMR (zero-excluded) |  |  | 21 | 15 |  |  |  |  |  |  |  |  |
|  | **Gassire** | Average camp population |  |  |  |  |  | 17,813 |  |  |  |  |  |  |
|  |  | Total Number of live births |  |  |  |  |  | 3,010 |  |  |  |  |  |  |
|  |  | # of months (including zero entries) |  |  |  |  |  | 6 |  |  |  |  |  |  |
|  |  | Average NMR (Zero-included) |  |  |  |  |  | 0 |  |  |  |  |  |  |
|  |  | # of months (excluding zero entries) |  |  |  |  |  |  |  |  |  |  |  |  |
|  |  | Average NMR (zero-excluded) |  |  |  |  |  |  |  |  |  |  |  |  |
|  | **Gondje** | Average camp population |  |  | 18,090 | 12,667 |  | 9,591 | 10,765 | 11,751 |  | 11,353 | 7,621 |  |
|  |  | Total Number of live births |  |  | 187 | 230 |  | 131 | 191 | 163 |  | 125 | 181 |  |
|  |  | # of months (including zero entries) |  |  | 9 | 10 |  | 7 | 9 | 9 |  | 7 | 8 |  |
|  |  | Average NMR (Zero-included) |  |  | 11 | 0 |  | 0 | 0 | 0 |  | 19 | 0 |  |
|  |  | # of months (excluding zero entries) |  |  | 2 |  |  |  |  |  |  | 2 |  |  |
|  |  | Average NMR (zero-excluded) |  |  | 48 |  |  |  |  |  |  | 67 |  |  |
|  | **Gouroukoune** | Average camp population |  |  |  |  |  | 18,112 |  |  |  |  |  |  |
|  |  | Total Number of live births |  |  |  |  |  | 3,133 |  |  |  |  |  |  |
|  |  | # of months (including zero entries) |  |  |  |  |  | 7 |  |  |  |  |  |  |
|  |  | Average NMR (Zero-included) |  |  |  |  |  | 0 |  |  |  |  |  |  |
|  |  | # of months (excluding zero entries) |  |  |  |  |  |  |  |  |  |  |  |  |
|  |  | Average NMR (zero-excluded) |  |  |  |  |  |  |  |  |  |  |  |  |
|  | **Goz Amer** | Average camp population |  |  | 20,532 | 22,074 | 22,542 | 24,509 | 26,512 | 28,529 | 30,404 | 31,365 | 31,521 | 32,991 |
|  |  | Total Number of live births |  |  | 957 | 926 | 1,057 | 1,073 | 1,017 | 884 | 9,338 | 1,344 | 1,256 | 975 |
|  |  | # of months (including zero entries) |  |  | 12 | 12 | 10 | 11 | 10 | 9 | 11 | 12 | 12 | 9 |
|  |  | Average NMR (Zero-included) |  |  | 1 | 1 | 1 | 0 | 5 | 0 | 0 | 0 | 0 | 0 |
|  |  | # of months (excluding zero entries) |  |  | 1 | 1 | 1 |  | 3 |  |  |  |  |  |
|  |  | Average NMR (zero-excluded) |  |  | 15 | 17 | 9 |  | 13 |  |  |  |  |  |
|  | **Iridimi** | Average camp population |  |  |  |  | 18,675 | 18,675 | 20,303 | 21,226 | 22,313 | 21,222 | 18,131 | 20,431 |
|  |  | Total Number of live births |  |  |  |  | 389 | 601 | 594 | 579 | 631 | 698 | 553 | 356 |
|  |  | # of months (including zero entries) |  |  |  |  | 7 | 11 | 11 | 12 | 12 | 12 | 12 | 9 |
|  |  | Average NMR (Zero-included) |  |  |  |  | 0 | 10 | 4 | 2 | 0 | 0 | 0 | 0 |
|  |  | # of months (excluding zero entries) |  |  |  |  |  | 3 | 3 | 1 |  |  |  |  |
|  |  | Average NMR (zero-excluded) |  |  |  |  |  | 31 | 16 | 24 |  |  |  |  |
|  | **Koloma** | Average camp population |  |  |  |  |  | 7,146 |  |  |  |  |  |  |
|  |  | Total Number of live births |  |  |  |  |  | 959 |  |  |  |  |  |  |
|  |  | # of months (including zero entries) |  |  |  |  |  | 7 |  |  |  |  |  |  |
|  |  | Average NMR (Zero-included) |  |  |  |  |  | 0 |  |  |  |  |  |  |
|  |  | # of months (excluding zero entries) |  |  |  |  |  |  |  |  |  |  |  |  |
|  |  | Average NMR (zero-excluded) |  |  |  |  |  |  |  |  |  |  |  |  |
|  | **Koubigou** | Average camp population |  |  |  |  |  | 12,521 |  |  |  |  |  |  |
|  |  | Total Number of live births |  |  |  |  |  | 2,106 |  |  |  |  |  |  |
|  |  | # of months (including zero entries) |  |  |  |  |  | 6 |  |  |  |  |  |  |
|  |  | Average NMR (Zero-included) |  |  |  |  |  | 0 |  |  |  |  |  |  |
|  |  | # of months (excluding zero entries) |  |  |  |  |  |  |  |  |  |  |  |  |
|  |  | Average NMR (zero-excluded) |  |  |  |  |  |  |  |  |  |  |  |  |
|  | **Kounoungou** | Average camp population |  |  | 16,746 | 18,644 | 17,162 | 16,953 | 18,751 | 17,828 | 20,966 | 19,260 | 19,705 | 21,637 |
|  |  | Total Number of live births |  |  | 623 | 625 | 502 | 447 | 684 | 651 | 657 | 688 | 7,993 | 3,854 |
|  |  | # of months (including zero entries) |  |  | 12 | 12 | 12 | 9 | 11 | 11 | 12 | 12 | 12 | 9 |
|  |  | Average NMR (Zero-included) |  |  | 4 | 13 | 9 | 0 | 3 | 0 | 0 | 0 | 0 | 0 |
|  |  | # of months (excluding zero entries) |  |  | 2 | 4 | 3 |  | 2 |  |  |  |  |  |
|  |  | Average NMR (zero-excluded) |  |  | 24 | 39 | 34 |  | 14 |  |  |  |  |  |
|  | **Mile** | Average camp population |  |  | 16,583 | 16,976 | 15,243 | 17,569 | 19,235 | 19,560 | 20,933 | 21,095 | 19,273 | 21,734 |
|  |  | Total Number of live births |  |  | 559 | 549 | 654 | 499 | 617 | 631 | 697 | 6,893 | 6,156 | 1,741 |
|  |  | # of months (including zero entries) |  |  | 12 | 12 | 11 | 9 | 10 | 10 | 12 | 12 | 12 | 8 |
|  |  | Average NMR (Zero-included) |  |  | 2 | 0 | 1 | 5 | 3 | 0 | 3 | 0 | 2 | 0 |
|  |  | # of months (excluding zero entries) |  |  |  |  | 1 | 3 | 2 |  | 2 |  | 2 |  |
|  |  | Average NMR (zero-excluded) |  |  |  |  | 14 | 16 | 13 |  | 16 |  | 10 |  |
|  | **Moula** | Average camp population |  |  |  | 5,501 | 5,251 | 5,304 | 5,911 |  |  |  |  |  |
|  |  | Total Number of live births |  |  |  | 257 | 148 | 178 | 136 |  |  |  |  |  |
|  |  | # of months (including zero entries) |  |  |  | 11 | 7 | 9 | 7 |  |  |  |  |  |
|  |  | Average NMR (Zero-included) |  |  |  | 0 | 12 | 6 | 0 |  |  |  |  |  |
|  |  | # of months (excluding zero entries) |  |  |  |  | 2 | 1 |  |  |  |  |  |  |
|  |  | Average NMR (zero-excluded) |  |  |  |  | 43 | 56 |  |  |  |  |  |  |
|  | **Moyo** | Average camp population |  |  |  |  |  | 3,555 | 6,637 | 8,432 | 9,240 | 9,627 | 7,979 | 7,845 |
|  |  | Total Number of live births |  |  |  |  |  | 123 | 248 | 184 | 232 | 218 | 190 | 193 |
|  |  | # of months (including zero entries) |  |  |  |  |  | 6 | 10 | 7 | 11 | 10 | 9 | 9 |
|  |  | Average NMR (Zero-included) |  |  |  |  |  | 0 | 0 | 17 | 9 | 0 | 7 | 0 |
|  |  | # of months (excluding zero entries) |  |  |  |  |  |  |  | 3 | 1 |  | 1 |  |
|  |  | Average NMR (zero-excluded) |  |  |  |  |  |  |  | 41 | 45 |  | 59 |  |
|  | **Oure Cassoni** | Average camp population |  |  | 27,424 | 27,691 | 31,208 | 32,826 | 32,261 | 33,552 | 35,596 | 32,715 | 28,020 | 27,858 |
|  |  | Total Number of live births |  |  | 709 | 917 | 818 | 957 | 875 | 731 | 840 | 864 | 863 | 688 |
|  |  | # of months (including zero entries) |  |  | 12 | 12 | 12 | 12 | 11 | 10 | 11 | 12 | 12 | 8 |
|  |  | Average NMR (Zero-included) |  |  | 12 | 10 | 18 | 28 | 16 | 14 | 2 | 2 | 0 | 1 |
|  |  | # of months (excluding zero entries) |  |  | 7 | 9 | 8 | 11 | 8 | 7 | 1 | 1 |  | 1 |
|  |  | Average NMR (zero-excluded) |  |  | 21 | 13 | 27 | 30 | 22 | 20 | 23 | 27 |  | 12 |
|  | **Touloum** | Average camp population |  |  |  |  |  | 23,066 | 23,978 | 28,359 | 29,240 | 26,398 | 22,267 | 22,099 |
|  |  | Total Number of live births |  |  |  |  |  | 755 | 721 | 977 | 913 | 987 | 791 | 479 |
|  |  | # of months (including zero entries) |  |  |  |  |  | 12 | 12 | 12 | 12 | 12 | 12 | 9 |
|  |  | Average NMR (Zero-included) |  |  |  |  |  | 8 | 2 | 1 | 4 | 2 | 1 | 6 |
|  |  | # of months (excluding zero entries) |  |  |  |  |  | 3 | 1 | 1 | 2 | 2 | 1 | 2 |
|  |  | Average NMR (zero-excluded) |  |  |  |  |  | 32 | 14 | 15 | 26 | 12 | 13 | 18 |
|  | **Treguine** | Average camp population |  |  | 16,137 | 16,944 | 17,054 | 18,299 | 19,472 | 20,523 | 21,284 | 21,433 | 21,986 | 22,811 |
|  |  | Total Number of live births |  |  | 889 | 1,168 | 852 | 950 | 884 | 475 | 769 | 718 | 756 | 382 |
|  |  | # of months (including zero entries) |  |  | 12 | 11 | 12 | 12 | 12 | 7 | 12 | 11 | 12 | 6 |
|  |  | Average NMR (Zero-included) |  |  | 0 | 6 | 1 | 6 | 6 | 3 | 3 | 2 | 1 | 0 |
|  |  | # of months (excluding zero entries) |  |  |  | 4 | 1 | 5 | 4 | 1 | 2 | 1 | 1 |  |
|  |  | Average NMR (zero-excluded) |  |  |  | 17 | 14 | 15 | 17 | 19 | 18 | 17 | 14 |  |
|  | **Yaroungou** | Average camp population |  |  | 13,337 |  | 10,674 | 13,038 | 11,393 |  |  |  |  |  |
|  |  | Total Number of live births |  |  | 148 |  | 305 | 275 | 299 |  |  |  |  |  |
|  |  | # of months (including zero entries) |  |  | 6 |  | 11 | 8 | 10 |  |  |  |  |  |
|  |  | Average NMR (Zero-included) |  |  | 0 |  | 26 | 16 | 14 |  |  |  |  |  |
|  |  | # of months (excluding zero entries) |  |  |  |  | 5 | 2 | 3 |  |  |  |  |  |
|  |  | Average NMR (zero-excluded) |  |  |  |  | 57 | 65 | 46 |  |  |  |  |  |
| **Democratic Republic of Congo** | **Bili** | Average camp population |  |  |  |  |  |  |  |  |  |  | 9,998 | 9,415 |
|  |  | Total Number of live births |  |  |  |  |  |  |  |  |  |  | 287 | 204 |
|  |  | # of months (including zero entries) |  |  |  |  |  |  |  |  |  |  | 12 | 8 |
|  |  | Average NMR (Zero-included) |  |  |  |  |  |  |  |  |  |  | 0 | 0 |
|  |  | # of months (excluding zero entries) |  |  |  |  |  |  |  |  |  |  |  |  |
|  |  | Average NMR (zero-excluded) |  |  |  |  |  |  |  |  |  |  |  |  |
|  | **Boyabo** | Average camp population |  |  |  |  |  |  |  |  | 11,128 | 17,733 | 18,516 | 17,835 |
|  |  | Total Number of live births |  |  |  |  |  |  |  |  | 378 | 402 | 472 | 341 |
|  |  | # of months (including zero entries) |  |  |  |  |  |  |  |  | 12 | 11 | 12 | 8 |
|  |  | Average NMR (Zero-included) |  |  |  |  |  |  |  |  | 3 | 13 | 9 | 9 |
|  |  | # of months (excluding zero entries) |  |  |  |  |  |  |  |  | 1 | 4 | 3 | 1 |
|  |  | Average NMR (zero-excluded) |  |  |  |  |  |  |  |  | 33 | 36 | 37 | 43 |
|  | **Inke** | Average camp population |  |  |  |  |  |  |  |  | 10,487 | 17,655 | 19,238 | 15,001 |
|  |  | Total Number of live births |  |  |  |  |  |  |  |  | 264 | 499 | 268 | 272 |
|  |  | # of months (including zero entries) |  |  |  |  |  |  |  |  | 9 | 12 | 9 | 8 |
|  |  | Average NMR (Zero-included) |  |  |  |  |  |  |  |  | 27 | 20 | 22 | 11 |
|  |  | # of months (excluding zero entries) |  |  |  |  |  |  |  |  | 5 | 5 | 3 | 2 |
|  |  | Average NMR (zero-excluded) |  |  |  |  |  |  |  |  | 49 | 49 | 66 | 42 |
|  | **Lusenda** | Average camp population |  |  |  |  |  |  |  |  |  |  | 18,631 | 27,713 |
|  |  | Total Number of live births |  |  |  |  |  |  |  |  |  |  | 919 | 963 |
|  |  | # of months (including zero entries) |  |  |  |  |  |  |  |  |  |  | 12 | 8 |
|  |  | Average NMR (Zero-included) |  |  |  |  |  |  |  |  |  |  | 1 | 6 |
|  |  | # of months (excluding zero entries) |  |  |  |  |  |  |  |  |  |  | 1 | 3 |
|  |  | Average NMR (zero-excluded) |  |  |  |  |  |  |  |  |  |  | 9 | 16 |
|  | **Mole** | Average camp population |  |  |  |  |  |  |  |  | 13,020 | 17,316 | 20,272 | 15,301 |
|  |  | Total Number of live births |  |  |  |  |  |  |  |  | 197 | 309 | 308 | 212 |
|  |  | # of months (including zero entries) |  |  |  |  |  |  |  |  | 10 | 12 | 12 | 8 |
|  |  | Average NMR (Zero-included) |  |  |  |  |  |  |  |  | 16 | 20 | 13 | 0 |
|  |  | # of months (excluding zero entries) |  |  |  |  |  |  |  |  | 3 | 4 | 4 |  |
|  |  | Average NMR (zero-excluded) |  |  |  |  |  |  |  |  | 53 | 60 | 40 |  |
| **Republic of Congo** | **Betou** | Average camp population |  |  |  |  |  |  |  | 19,204 | 17,752 |  |  |  |
|  |  | Total Number of live births |  |  |  |  |  |  |  | 619 | 469 |  |  |  |
|  |  | # of months (including zero entries) |  |  |  |  |  |  |  | 12 | 6 |  |  |  |
|  |  | Average NMR (Zero-included) |  |  |  |  |  |  |  | 8 | 14 |  |  |  |
|  |  | # of months (excluding zero entries) |  |  |  |  |  |  |  | 5 | 4 |  |  |  |
|  |  | Average NMR (zero-excluded) |  |  |  |  |  |  |  | 19 | 21 |  |  |  |
|  | **Impfondo** | Average camp population |  |  |  |  |  |  | 58,157 | 36,191 |  |  |  |  |
|  |  | Total Number of live births |  |  |  |  |  |  | 1,258 | 539 |  |  |  |  |
|  |  | # of months (including zero entries) |  |  |  |  |  |  | 11 | 8 |  |  |  |  |
|  |  | Average NMR (Zero-included) |  |  |  |  |  |  | 23 | 14 |  |  |  |  |
|  |  | # of months (excluding zero entries) |  |  |  |  |  |  | 9 | 4 |  |  |  |  |
|  |  | Average NMR (zero-excluded) |  |  |  |  |  |  | 27 | 29 |  |  |  |  |
| **Djibouti** | **Ali Adde** | Average camp population |  |  | 7,657 | 9,775 | 12,700 | 16,921 | 16,104 | 17,398 | 17,809 | 10,600 | 11,345 | 14,035 |
|  |  | Total Number of live births |  |  | 125 | 223 | 258 | 290 | 341 | 281 | 295 | 216 | 244 | 128 |
|  |  | # of months (including zero entries) |  |  | 7 | 10 | 11 | 10 | 12 | 11 | 11 | 10 | 9 | 6 |
|  |  | Average NMR (Zero-included) |  |  | 0 | 8 | 4 | 6 | 3 | 5 | 8 | 4 | 0 | 0 |
|  |  | # of months (excluding zero entries) |  |  |  | 2 | 1 | 2 | 1 | 1 | 2 | 1 |  |  |
|  |  | Average NMR (zero-excluded) |  |  |  | 42 | 40 | 32 | 33 | 53 | 46 | 38 |  |  |
| **Ethiopia** | **Awbarre** | Average camp population |  |  | 10,040 | 10,416 | 12,966 | 12,794 | 13,329 | 13,412 | 12,481 | 12,827 | 12,399 | 10,447 |
|  |  | Total Number of live births |  |  | 184 | 220 | 322 | 307 | 234 | 450 | 403 | 387 | 363 | 165 |
|  |  | # of months (including zero entries) |  |  | 8 | 9 | 11 | 11 | 9 | 11 | 12 | 12 | 12 | 6 |
|  |  | Average NMR (Zero-included) |  |  | 0 | 0 | 0 | 0 | 0 | 0 | 3 | 0 | 0 | 0 |
|  |  | # of months (excluding zero entries) |  |  |  |  |  |  |  |  | 1 |  |  |  |
|  |  | Average NMR (zero-excluded) |  |  |  |  |  |  |  |  | 33 |  |  |  |
|  | **Aysaita** | Average camp population |  |  |  |  |  |  |  |  | 8,383 |  |  |  |
|  |  | Total Number of live births |  |  |  |  |  |  |  |  | 4,684 |  |  |  |
|  |  | # of months (including zero entries) |  |  |  |  |  |  |  |  | 7 |  |  |  |
|  |  | Average NMR (Zero-included) |  |  |  |  |  |  |  |  | 9 |  |  |  |
|  |  | # of months (excluding zero entries) |  |  |  |  |  |  |  |  | 1 |  |  |  |
|  |  | Average NMR (zero-excluded) |  |  |  |  |  |  |  |  | 63 |  |  |  |
|  | **Bambasi** | Average camp population |  |  |  |  |  |  |  | 12,794 | 13,626 | 13,854 | 14,876 | 14,849 |
|  |  | Total Number of live births |  |  |  |  |  |  |  | 978 | 549 | 917 | 656 | 607 |
|  |  | # of months (including zero entries) |  |  |  |  |  |  |  | 9 | 12 | 12 | 10 | 8 |
|  |  | Average NMR (Zero-included) |  |  |  |  |  |  |  | 4 | 0 | 11 | 0 | 0 |
|  |  | # of months (excluding zero entries) |  |  |  |  |  |  |  | 1 |  | 4 |  |  |
|  |  | Average NMR (zero-excluded) |  |  |  |  |  |  |  | 32 |  | 33 |  |  |
|  | **Barahle** | Average camp population |  |  |  |  |  |  |  |  |  |  | 9,650 |  |
|  |  | Total Number of live births |  |  |  |  |  |  |  |  |  |  | 162 |  |
|  |  | # of months (including zero entries) |  |  |  |  |  |  |  |  |  |  | 8 |  |
|  |  | Average NMR (Zero-included) |  |  |  |  |  |  |  |  |  |  | 0 |  |
|  |  | # of months (excluding zero entries) |  |  |  |  |  |  |  |  |  |  |  |  |
|  |  | Average NMR (zero-excluded) |  |  |  |  |  |  |  |  |  |  |  |  |
|  | **Bokolmanyo** | Average camp population |  |  |  |  | 16,438 | 31,914 | 39,529 | 41,459 | 41,858 | 42,071 | 42,385 | 42,385 |
|  |  | Total Number of live births |  |  |  |  | 353 | 698 | 655 | 489 | 537 | 635 | 554 | 365 |
|  |  | # of months (including zero entries) |  |  |  |  | 11 | 12 | 12 | 11 | 12 | 12 | 11 | 7 |
|  |  | Average NMR (Zero-included) |  |  |  |  | 3 | 0 | 5 | 2 | 0 | 0 | 0 | 0 |
|  |  | # of months (excluding zero entries) |  |  |  |  | 1 |  | 2 | 1 |  |  |  |  |
|  |  | Average NMR (zero-excluded) |  |  |  |  | 31 |  | 30 | 22 |  |  |  |  |
|  | **Bonga** | Average camp population |  | 8940 |  |  |  |  |  |  |  |  |  |  |
|  |  | Total Number of live births |  | 388 |  |  |  |  |  |  |  |  |  |  |
|  |  | # of months (including zero entries) |  | 11 |  |  |  |  |  |  |  |  |  |  |
|  |  | Average NMR (Zero-included) |  | 5 |  |  |  |  |  |  |  |  |  |  |
|  |  | # of months (excluding zero entries) |  | 1 |  |  |  |  |  |  |  |  |  |  |
|  |  | Average NMR (zero-excluded) |  | 52.63 |  |  |  |  |  |  |  |  |  |  |
|  | **Buramino** | Average camp population |  |  |  |  |  |  |  | 39,219 | 39,825 | 39,060 | 24,206 | 29,022 |
|  |  | Total Number of live births |  |  |  |  |  |  |  | 599 | 591 | 489 | 445 | 371 |
|  |  | # of months (including zero entries) |  |  |  |  |  |  |  | 12 | 12 | 12 | 10 | 8 |
|  |  | Average NMR (Zero-included) |  |  |  |  |  |  |  | 0 | 0 | 0 | 0 | 0 |
|  |  | # of months (excluding zero entries) |  |  |  |  |  |  |  |  |  |  |  |  |
|  |  | Average NMR (zero-excluded) |  |  |  |  |  |  |  |  |  |  |  |  |
|  | **Dimma** | Average camp population |  | 5178 |  |  |  |  |  |  |  |  |  |  |
|  |  | Total Number of live births |  | 125 |  |  |  |  |  |  |  |  |  |  |
|  |  | # of months (including zero entries) |  | 6 |  |  |  |  |  |  |  |  |  |  |
|  |  | Average NMR (Zero-included) |  | 0 |  |  |  |  |  |  |  |  |  |  |
|  |  | # of months (excluding zero entries) |  |  |  |  |  |  |  |  |  |  |  |  |
|  |  | Average NMR (zero-excluded) |  |  |  |  |  |  |  |  |  |  |  |  |
|  | **Fugnido** | Average camp population |  | 22692 | 20,696 | 21,314 | 21,221 | 22,133 | 26,790 | 38,386 | 44,278 | 59,930 | 59,251 | 64,819 |
|  |  | Total Number of live births |  | 936 | 986 | 1,159 | 1,335 | 476 | 912 | 1,580 | 1,799 | 2,414 | 1,795 | 1,019 |
|  |  | # of months (including zero entries) |  | 12 | 12 | 12 | 12 | 6 | 10 | 12 | 12 | 12 | 12 | 6 |
|  |  | Average NMR (Zero-included) |  | 0 | 2 | 2 | 0 | 0 | 0 | 0 | 0 | 0 | 0 | 0 |
|  |  | # of months (excluding zero entries) |  |  | 1 | 2 |  |  |  |  |  |  |  |  |
|  |  | Average NMR (zero-excluded) |  |  | 12 | 10 |  |  |  |  |  |  |  |  |
|  | **Fugnido 2** | Average camp population |  |  |  |  |  |  |  |  |  |  | 16,331 | 16,880 |
|  |  | Total Number of live births |  |  |  |  |  |  |  |  |  |  | 230 | 289 |
|  |  | # of months (including zero entries) |  |  |  |  |  |  |  |  |  |  | 8 | 8 |
|  |  | Average NMR (Zero-included) |  |  |  |  |  |  |  |  |  |  | 0 | 0 |
|  |  | # of months (excluding zero entries) |  |  |  |  |  |  |  |  |  |  |  |  |
|  |  | Average NMR (zero-excluded) |  |  |  |  |  |  |  |  |  |  |  |  |
|  | **Hilaweyn** | Average camp population |  |  |  |  |  |  |  | 35,100 | 39,072 | 39,000 | 43,169 | 43,938 |
|  |  | Total Number of live births |  |  |  |  |  |  |  | 271 | 376 | 581 | 474 | 573 |
|  |  | # of months (including zero entries) |  |  |  |  |  |  |  | 9 | 10 | 11 | 9 | 8 |
|  |  | Average NMR (Zero-included) |  |  |  |  |  |  |  | 0 | 8 | 0 | 0 | 0 |
|  |  | # of months (excluding zero entries) |  |  |  |  |  |  |  |  | 2 |  |  |  |
|  |  | Average NMR (zero-excluded) |  |  |  |  |  |  |  |  | 40 |  |  |  |
|  | **Jewi** | Average camp population |  |  |  |  |  |  |  |  |  |  | 46,834 | 57,819 |
|  |  | Total Number of live births |  |  |  |  |  |  |  |  |  |  | 905 | 1,152 |
|  |  | # of months (including zero entries) |  |  |  |  |  |  |  |  |  |  | 11 | 9 |
|  |  | Average NMR (Zero-included) |  |  |  |  |  |  |  |  |  |  | 1 | 0 |
|  |  | # of months (excluding zero entries) |  |  |  |  |  |  |  |  |  |  | 1 |  |
|  |  | Average NMR (zero-excluded) |  |  |  |  |  |  |  |  |  |  | 12 |  |
|  | **Kebribeyah** | Average camp population |  | 16614 | 16,220 | 16,362 | 15,973 | 16,695 | 16,219 | 15,921 | 15,354 | 14,556 | 13,614 | 14,224 |
|  |  | Total Number of live births |  | 553 | 484 | 453 | 648 | 663 | 506 | 742 | 743 | 736 | 710 | 495 |
|  |  | # of months (including zero entries) |  | 12 | 11 | 11 | 12 | 11 | 9 | 12 | 12 | 12 | 12 | 8 |
|  |  | Average NMR (Zero-included) |  | 0 | 6 | 2 | 2 | 0 | 0 | 3 | 1 | 0 | 0 | 4 |
|  |  | # of months (excluding zero entries) |  |  | 1 | 1 | 1 |  |  | 2 | 1 |  |  | 1 |
|  |  | Average NMR (zero-excluded) |  |  | 43 | 23 | 22 |  |  | 17 | 16 |  |  | 33 |
|  | **Kobe** | Average camp population |  |  |  |  |  |  | 27,619 | 35,404 | 37,811 | 40,719 | 41,491 | 38,116 |
|  |  | Total Number of live births |  |  |  |  |  |  | 247 | 872 | 703 | 813 | 705 | 579 |
|  |  | # of months (including zero entries) |  |  |  |  |  |  | 6 | 12 | 12 | 12 | 10 | 7 |
|  |  | Average NMR (Zero-included) |  |  |  |  |  |  | 0 | 5 | 2 | 0 | 0 | 0 |
|  |  | # of months (excluding zero entries) |  |  |  |  |  |  |  | 3 | 1 |  |  |  |
|  |  | Average NMR (zero-excluded) |  |  |  |  |  |  |  | 18 | 24 |  |  |  |
|  | **Kule** | Average camp population |  |  |  |  |  |  |  |  | 44,392 | 47,288 | 48,703 | 51,289 |
|  |  | Total Number of live births |  |  |  |  |  |  |  |  | 550 | 1,160 | 1,221 | 933 |
|  |  | # of months (including zero entries) |  |  |  |  |  |  |  |  | 7 | 10 | 12 | 8 |
|  |  | Average NMR (Zero-included) |  |  |  |  |  |  |  |  | 0 | 7 | 1 | 0 |
|  |  | # of months (excluding zero entries) |  |  |  |  |  |  |  |  |  | 5 | 1 |  |
|  |  | Average NMR (zero-excluded) |  |  |  |  |  |  |  |  |  | 14 | 12 |  |
|  | **Leitchuor** | Average camp population |  |  |  |  |  |  |  |  | 48,011 | 47,964 |  |  |
|  |  | Total Number of live births |  |  |  |  |  |  |  |  | 645 | 886 |  |  |
|  |  | # of months (including zero entries) |  |  |  |  |  |  |  |  | 7 | 6 |  |  |
|  |  | Average NMR (Zero-included) |  |  |  |  |  |  |  |  | 4 | 9 |  |  |
|  |  | # of months (excluding zero entries) |  |  |  |  |  |  |  |  | 3 | 4 |  |  |
|  |  | Average NMR (zero-excluded) |  |  |  |  |  |  |  |  | 10 | 14 |  |  |
|  | **Mai Aini** | Average camp population |  |  |  |  |  | 12,967 | 14,381 | 15,711 | 18,616 |  | 20,629 |  |
|  |  | Total Number of live births |  |  |  |  |  | 279 | 242 | 242 | 195 |  | 150 |  |
|  |  | # of months (including zero entries) |  |  |  |  |  | 11 | 10 | 12 | 10 |  | 8 |  |
|  |  | Average NMR (Zero-included) |  |  |  |  |  | 3 | 0 | 3 | 5 |  | 6 |  |
|  |  | # of months (excluding zero entries) |  |  |  |  |  | 1 |  | 1 | 1 |  | 1 |  |
|  |  | Average NMR (zero-excluded) |  |  |  |  |  | 38 |  | 40 | 50 |  | 50 |  |
|  | **Melkadida** | Average camp population |  |  |  |  |  | 35,610 | 41,170 | 43,498 | 43,992 | 44,671 | 44,671 | 43,326 |
|  |  | Total Number of live births |  |  |  |  |  | 805 | 683 | 649 | 530 | 538 | 629 | 454 |
|  |  | # of months (including zero entries) |  |  |  |  |  | 12 | 11 | 12 | 12 | 11 | 10 | 8 |
|  |  | Average NMR (Zero-included) |  |  |  |  |  | 1 | 14 | 2 | 0 | 0 | 0 | 0 |
|  |  | # of months (excluding zero entries) |  |  |  |  |  | 1 | 4 | 1 |  |  |  |  |
|  |  | Average NMR (zero-excluded) |  |  |  |  |  | 13 | 39 | 28 |  |  |  |  |
|  | **Nguenyyiel** | Average camp population |  |  |  |  |  |  |  |  |  |  |  | 53,505 |
|  |  | Total Number of live births |  |  |  |  |  |  |  |  |  |  |  | 561 |
|  |  | # of months (including zero entries) |  |  |  |  |  |  |  |  |  |  |  | 6 |
|  |  | Average NMR (Zero-included) |  |  |  |  |  |  |  |  |  |  |  | 0 |
|  |  | # of months (excluding zero entries) |  |  |  |  |  |  |  |  |  |  |  |  |
|  |  | Average NMR (zero-excluded) |  |  |  |  |  |  |  |  |  |  |  |  |
|  | **Okugo** | Average camp population |  |  |  |  |  |  |  |  |  | 6,789 | 8,236 | 11,061 |
|  |  | Total Number of live births |  |  |  |  |  |  |  |  |  | 240 | 215 | 191 |
|  |  | # of months (including zero entries) |  |  |  |  |  |  |  |  |  | 10 | 9 | 7 |
|  |  | Average NMR (Zero-included) |  |  |  |  |  |  |  |  |  | 6 | 0 | 0 |
|  |  | # of months (excluding zero entries) |  |  |  |  |  |  |  |  |  | 1 |  |  |
|  |  | Average NMR (zero-excluded) |  |  |  |  |  |  |  |  |  | 63 |  |  |
|  | **Sheder** | Average camp population |  |  |  |  | 9,441 | 11,247 | 11,637 | 11,919 | 11,962 | 11,861 | 11,244 | 10,890 |
|  |  | Total Number of live births |  |  |  |  | 181 | 276 | 1,069 | 228 | 207 | 396 | 332 | 218 |
|  |  | # of months (including zero entries) |  |  |  |  | 8 | 11 | 11 | 10 | 8 | 12 | 12 | 8 |
|  |  | Average NMR (Zero-included) |  |  |  |  | 0 | 0 | 0 | 4 | 0 | 0 | 3 | 0 |
|  |  | # of months (excluding zero entries) |  |  |  |  |  |  |  | 1 |  |  | 1 |  |
|  |  | Average NMR (zero-excluded) |  |  |  |  |  |  |  | 38 |  |  | 40 |  |
|  | **Sherkole** | Average camp population |  | 11022 |  |  |  |  | 9,437 |  | 19,829 | 14,441 | 11,538 | 11,529 |
|  |  | Total Number of live births |  | 303 |  |  |  |  | 293 |  | 625 | 601 | 549 | 308 |
|  |  | # of months (including zero entries) |  | 11 |  |  |  |  | 11 |  | 11 | 12 | 12 | 8 |
|  |  | Average NMR (Zero-included) |  | 0 |  |  |  |  | 0 |  | 3 | 5 | 7 | 7 |
|  |  | # of months (excluding zero entries) |  |  |  |  |  |  |  |  | 1 | 2 | 2 | 2 |
|  |  | Average NMR (zero-excluded) |  |  |  |  |  |  |  |  | 31 | 18 | 40 | 28 |
|  | **Shimelba** | Average camp population |  | 14,691 | 15,210 | 10,326 | 9,193 | 7,786 | 7,548 |  |  |  |  |  |
|  |  | Total Number of live births |  | 261 | 301 | 187 | 196 | 166 | 101 |  |  |  |  |  |
|  |  | # of months (including zero entries) |  | 11 | 11 | 9 | 9 | 7 | 6 |  |  |  |  |  |
|  |  | Average NMR (Zero-included) |  | 3 | 6 | 32 | 0 | 0 | 10 |  |  |  |  |  |
|  |  | # of months (excluding zero entries) |  | 1 | 1 | 3 |  |  | 1 |  |  |  |  |  |
|  |  | Average NMR (zero-excluded) |  | 38.46 | 37 | 65 |  |  | 63 |  |  |  |  |  |
|  | **Tierkidi** | Average camp population |  |  |  |  |  |  |  |  | 47,405 | 49,518 | 52,472 | 67,510 |
|  |  | Total Number of live births |  |  |  |  |  |  |  |  | 916 | 1,601 | 1,415 | 1,343 |
|  |  | # of months (including zero entries) |  |  |  |  |  |  |  |  | 8 | 11 | 10 | 8 |
|  |  | Average NMR (Zero-included) |  |  |  |  |  |  |  |  | 0 | 3 | 2 | 0 |
|  |  | # of months (excluding zero entries) |  |  |  |  |  |  |  |  |  | 4 | 2 |  |
|  |  | Average NMR (zero-excluded) |  |  |  |  |  |  |  |  |  | 7 | 8 |  |
|  | **Tongo** | Average camp population |  |  |  |  |  |  | 12,612 | 9,577 | 10,898 | 11,490 | 11,595 | 12,170 |
|  |  | Total Number of live births |  |  |  |  |  |  | 306 | 432 | 406 | 493 | 501 | 259 |
|  |  | # of months (including zero entries) |  |  |  |  |  |  | 9 | 9 | 11 | 12 | 12 | 7 |
|  |  | Average NMR (Zero-included) |  |  |  |  |  |  | 0 | 2 | 3 | 3 | 0 | 16 |
|  |  | # of months (excluding zero entries) |  |  |  |  |  |  |  | 1 | 1 | 2 |  | 3 |
|  |  | Average NMR (zero-excluded) |  |  |  |  |  |  |  | 19 | 30 | 21 |  | 37 |
|  | **Tsore** | Average camp population |  |  |  |  |  |  |  |  |  |  |  | 11,125 |
|  |  | Total Number of live births |  |  |  |  |  |  |  |  |  |  |  | 285 |
|  |  | # of months (including zero entries) |  |  |  |  |  |  |  |  |  |  |  | 8 |
|  |  | Average NMR (Zero-included) |  |  |  |  |  |  |  |  |  |  |  | 0 |
|  |  | # of months (excluding zero entries) |  |  |  |  |  |  |  |  |  |  |  |  |
|  |  | Average NMR (zero-excluded) |  |  |  |  |  |  |  |  |  |  |  |  |
| **Kenya** | **Dagahaley** | Average camp population | 34924 | 38935 | 51,407 | 88,153 | 100,696 | 117,606 | 123,705 | 109,706 | 87,863 | 87,580 | 80,686 | 69,925 |
|  |  | Total Number of live births | 850 | 1015 | 1,731 | 2,056 | 2,296 | 3,553 | 3,382 | 3,211 | 2,931 | 2,945 | 2,861 | 2,296 |
|  |  | # of months (including zero entries) | 10 | 11 | 12 | 12 | 12 | 12 | 12 | 12 | 12 | 12 | 12 | 10 |
|  |  | Average NMR (Zero-included) | 5 | 2 | 3 | 1 | 2 | 8 | 10 | 14 | 10 | 11 | 13 | 10 |
|  |  | # of months (excluding zero entries) | 4 | 2 | 3 | 1 | 3 | 7 | 11 | 11 | 12 | 11 | 11 | 10 |
|  |  | Average NMR (zero-excluded) | 14 | 9 | 10 | 7 | 7 | 13 | 11 | 16 | 10 | 12 | 14 | 10 |
|  | **Hagadera** | Average camp population | 50,140 | 67,447 | 80,771 | 91,134 | 89,408 | 125,111 | 139,883 | 127,401 | 109,916 | 106,708 | 100,638 | 85,189 |
|  |  | Total Number of live births | 1703 | 1979 | 2,836 | 2,518 | 2,766 | 3,608 | 3,434 | 3,096 | 2,925 | 3,011 | 2,897 | 2,506 |
|  |  | # of months (including zero entries) | 11 | 11 | 12 | 12 | 12 | 12 | 12 | 12 | 12 | 12 | 12 | 10 |
|  |  | Average NMR (Zero-included) | 3 | 3 | 1 | 11 | 4 | 13 | 8 | 3 | 5 | 10 | 23 | 16 |
|  |  | # of months (excluding zero entries) | 4 | 4 | 3 | 10 | 6 | 11 | 11 | 4 | 7 | 6 | 12 | 10 |
|  |  | Average NMR (zero-excluded) | 8.11 | 8.125 | 4 | 14 | 8 | 14 | 9 | 9 | 9 | 20 | 23 | 16 |
|  | **Ifo** | Average camp population | 51,392 | 59,608 | 71,134 | 91,200 | 89,684 | 122,856 | 117,370 | 99,594 | 87,632 | 83,533 | 80,467 | 65,871 |
|  |  | Total Number of live births | 1026 | 1314 | 2,118 | 2,260 | 2,298 | 3,103 | 3,300 | 2,508 | 2,443 | 2,292 | 2,235 | 1,883 |
|  |  | # of months (including zero entries) | 10 | 11 | 12 | 12 | 12 | 12 | 12 | 12 | 12 | 12 | 12 | 10 |
|  |  | Average NMR (Zero-included) | 3 | 2 | 1 | 2 | 1 | 5 | 10 | 14 | 11 | 17 | 11 | 8 |
|  |  | # of months (excluding zero entries) | 2 | 1 | 3 | 2 | 2 | 6 | 11 | 12 | 10 | 11 | 11 | 8 |
|  |  | Average NMR (zero-excluded) | 13 | 25 | 5 | 11 | 5 | 11 | 11 | 14 | 13 | 18 | 12 | 10 |
|  | **Ifo 2** | Average camp population |  |  |  |  |  |  | 79,198 | 66,236 | 54,926 | 52,064 | 45,824 | 27,342 |
|  |  | Total Number of live births |  |  |  |  |  |  | 1,711 | 2,031 | 1,979 | 1,713 | 1,588 | 1,000 |
|  |  | # of months (including zero entries) |  |  |  |  |  |  | 8 | 12 | 12 | 12 | 12 | 10 |
|  |  | Average NMR (Zero-included) |  |  |  |  |  |  | 4 | 6 | 14 | 17 | 12 | 17 |
|  |  | # of months (excluding zero entries) |  |  |  |  |  |  | 4 | 7 | 12 | 11 | 7 | 7 |
|  |  | Average NMR (zero-excluded) |  |  |  |  |  |  | 7 | 10 | 14 | 19 | 20 | 24 |
|  | **Kakuma** | Average camp population | 92899 | 6584 | 53,305 | 50,451 | 72,807 | 82,097 | 97,161 | 114,417 | 160,037 | 182,225 | 181,037 | 142,504 |
|  |  | Total Number of live births | 2107 | 1599 | 1,377 | 1,651 | 2,260 | 2,543 | 2,825 | 3,073 | 4,485 | 3,909 | 3,755 | 3,862 |
|  |  | # of months (including zero entries) | 12 | 12 | 12 | 12 | 12 | 12 | 12 | 12 | 12 | 12 | 12 | 10 |
|  |  | Average NMR (Zero-included) | 12 | 2 | 3 | 6 | 9 | 7 | 7 | 4 | 5 | 4 | 3 | 3 |
|  |  | # of months (excluding zero entries) | 7 | 3 | 4 | 7 | 8 | 8 | 10 | 7 | 8 | 8 | 5 | 4 |
|  |  | Average NMR (zero-excluded) | 20 | 9 | 10 | 10 | 13 | 11 | 8 | 7 | 8 | 6 | 7 | 7 |
|  | **Kalobeyei** | Average camp population |  |  |  |  |  |  |  |  |  |  |  | 34,321 |
|  |  | Total Number of live births |  |  |  |  |  |  |  |  |  |  |  | 656 |
|  |  | # of months (including zero entries) |  |  |  |  |  |  |  |  |  |  |  | 8 |
|  |  | Average NMR (Zero-included) |  |  |  |  |  |  |  |  |  |  |  | 7 |
|  |  | # of months (excluding zero entries) |  |  |  |  |  |  |  |  |  |  |  | 4 |
|  |  | Average NMR (zero-excluded) |  |  |  |  |  |  |  |  |  |  |  | 13 |
|  | **Kambioos** | Average camp population |  |  |  |  |  |  | 14,413 | 19,144 | 20,186 | 20,593 | 17,195 |  |
|  |  | Total Number of live births |  |  |  |  |  |  | 286 | 396 | 440 | 569 | 601 |  |
|  |  | # of months (including zero entries) |  |  |  |  |  |  | 10 | 12 | 12 | 12 | 12 |  |
|  |  | Average NMR (Zero-included) |  |  |  |  |  |  | 0 | 4 | 0 | 7 | 0 |  |
|  |  | # of months (excluding zero entries) |  |  |  |  |  |  |  | 2 |  | 2 |  |  |
|  |  | Average NMR (zero-excluded) |  |  |  |  |  |  |  | 21 |  | 40 |  |  |
| **Liberia** | **Bahn** | Average camp population |  |  |  |  |  |  |  | 8,373 | 6,523 |  |  |  |
|  |  | Total Number of live births |  |  |  |  |  |  |  | 167 | 154 |  |  |  |
|  |  | # of months (including zero entries) |  |  |  |  |  |  |  | 7 | 8 |  |  |  |
|  |  | Average NMR (Zero-included) |  |  |  |  |  |  |  | 24 | 7 |  |  |  |
|  |  | # of months (excluding zero entries) |  |  |  |  |  |  |  | 4 | 1 |  |  |  |
|  |  | Average NMR (zero-excluded) |  |  |  |  |  |  |  | 42 | 59 |  |  |  |
|  | **PTP** | Average camp population |  |  |  |  |  |  |  | 11,422 | 15,135 | 15,389 | 9,520 |  |
|  |  | Total Number of live births |  |  |  |  |  |  |  | 656 | 237 | 423 | 317 |  |
|  |  | # of months (including zero entries) |  |  |  |  |  |  |  | 9 | 7 | 11 | 11 |  |
|  |  | Average NMR (Zero-included) |  |  |  |  |  |  |  | 0 | 0 | 7 | 2 |  |
|  |  | # of months (excluding zero entries) |  |  |  |  |  |  |  |  |  | 3 | 1 |  |
|  |  | Average NMR (zero-excluded) |  |  |  |  |  |  |  |  |  | 27 | 22 |  |
| **Nepal** | **Beldangi** | Average camp population |  |  |  |  |  |  | 34,265 | 28,214 | 21,092 | 15,897 |  |  |
|  |  | Total Number of live births |  |  |  |  |  |  | 672 | 501 | 348 | 223 |  |  |
|  |  | # of months (including zero entries) |  |  |  |  |  |  | 12 | 12 | 12 | 10 |  |  |
|  |  | Average NMR (Zero-included) |  |  |  |  |  |  | 6 | 14 | 7 | 8 |  |  |
|  |  | # of months (excluding zero entries) |  |  |  |  |  |  | 4 | 5 | 2 | 2 |  |  |
|  |  | Average NMR (zero-excluded) |  |  |  |  |  |  | 18 | 33 | 42 | 38 |  |  |
|  | **Beldangi I** | Average camp population |  |  | 18,311 | 16,428 | 13,917 |  |  |  |  |  |  |  |
|  |  | Total Number of live births |  |  | 289 | 313 | 224 |  |  |  |  |  |  |  |
|  |  | # of months (including zero entries) |  |  | 10 | 11 | 10 |  |  |  |  |  |  |  |
|  |  | Average NMR (Zero-included) |  |  | 5 | 3 | 0 |  |  |  |  |  |  |  |
|  |  | # of months (excluding zero entries) |  |  | 1 | 1 |  |  |  |  |  |  |  |  |
|  |  | Average NMR (zero-excluded) |  |  | 53 | 29 |  |  |  |  |  |  |  |  |
|  | **Beldangi II** | Average camp population |  |  | 22,377 | 19,620 | 15,913 |  |  |  |  |  |  |  |
|  |  | Total Number of live births |  |  | 301 | 358 | 238 |  |  |  |  |  |  |  |
|  |  | # of months (including zero entries) |  |  | 11 | 12 | 9 |  |  |  |  |  |  |  |
|  |  | Average NMR (Zero-included) |  |  | 0 | 7 | 0 |  |  |  |  |  |  |  |
|  |  | # of months (excluding zero entries) |  |  |  | 2 |  |  |  |  |  |  |  |  |
|  |  | Average NMR (zero-excluded) |  |  |  | 44 |  |  |  |  |  |  |  |  |
|  | **Beldangi II ext** | Average camp population |  |  | 11,634 | 10,966 |  |  |  |  |  |  |  |  |
|  |  | Total Number of live births |  |  | 153 | 136 |  |  |  |  |  |  |  |  |
|  |  | # of months (including zero entries) |  |  | 7 | 7 |  |  |  |  |  |  |  |  |
|  |  | Average NMR (Zero-included) |  |  | 0 | 8 |  |  |  |  |  |  |  |  |
|  |  | # of months (excluding zero entries) |  |  |  | 1 |  |  |  |  |  |  |  |  |
|  |  | Average NMR (zero-excluded) |  |  |  | 56 |  |  |  |  |  |  |  |  |
|  | **Khudunabari** | Average camp population |  |  | 13,252 | 12,429 | 11,375 |  |  |  |  |  |  |  |
|  |  | Total Number of live births |  |  | 133 | 172 | 167 |  |  |  |  |  |  |  |
|  |  | # of months (including zero entries) |  |  | 7 | 8 | 9 |  |  |  |  |  |  |  |
|  |  | Average NMR (Zero-included) |  |  | 0 | 0 | 0 |  |  |  |  |  |  |  |
|  |  | # of months (excluding zero entries) |  |  |  |  |  |  |  |  |  |  |  |  |
|  |  | Average NMR (zero-excluded) |  |  |  |  |  |  |  |  |  |  |  |  |
|  | **Sanishare** | Average camp population |  |  | 21,010 | 18,502 | 15,494 | 11,972 | 9,910 |  |  |  |  |  |
|  |  | Total Number of live births |  |  | 297 | 297 | 244 | 192 | 146 |  |  |  |  |  |
|  |  | # of months (including zero entries) |  |  | 10 | 11 | 10 | 9 | 7 |  |  |  |  |  |
|  |  | Average NMR (Zero-included) |  |  | 3 | 3 | 12 | 4 | 22 |  |  |  |  |  |
|  |  | # of months (excluding zero entries) |  |  | 1 | 1 | 2 | 1 | 3 |  |  |  |  |  |
|  |  | Average NMR (zero-excluded) |  |  | 34 | 38 | 62 | 38 | 51 |  |  |  |  |  |
|  | **Timai** | Average camp population |  |  | 10,343 | 9,186 |  |  |  |  |  |  |  |  |
|  |  | Total Number of live births |  |  | 138 | 174 |  |  |  |  |  |  |  |  |
|  |  | # of months (including zero entries) |  |  | 7 | 9 |  |  |  |  |  |  |  |  |
|  |  | Average NMR (Zero-included) |  |  | 21 | 0 |  |  |  |  |  |  |  |  |
|  |  | # of months (excluding zero entries) |  |  | 3 |  |  |  |  |  |  |  |  |  |
|  |  | Average NMR (zero-excluded) |  |  | 49 |  |  |  |  |  |  |  |  |  |
| **Rwanda** | **Gihembe** | Average camp population |  |  | 18,039 | 18,976 | 19,386 | 19,946 | 18,190 | 14,408 | 14,962 | 14,549 | 13,454 | 12,567 |
|  |  | Total Number of live births |  |  | 301 | 205 | 228 | 299 | 345 | 367 | 405 | 464 | 416 | 292 |
|  |  | # of months (including zero entries) |  |  | 8 | 6 | 7 | 10 | 12 | 12 | 12 | 12 | 12 | 9 |
|  |  | Average NMR (Zero-included) |  |  | 6 | 4 | 0 | 7 | 5 | 27 | 12 | 13 | 5 | 12 |
|  |  | # of months (excluding zero entries) |  |  | 2 | 1 |  | 2 | 1 | 7 | 5 | 5 | 2 | 3 |
|  |  | Average NMR (zero-excluded) |  |  | 24 | 25 |  | 36 | 25 | 46 | 29 | 31 | 29 | 35 |
|  | **Kigeme** | Average camp population |  |  |  |  |  |  |  |  | 18,428 | 18,614 | 19,143 | 19,718 |
|  |  | Total Number of live births |  |  |  |  |  |  |  |  | 518 | 594 | 578 | 467 |
|  |  | # of months (including zero entries) |  |  |  |  |  |  |  |  | 12 | 12 | 12 | 9 |
|  |  | Average NMR (Zero-included) |  |  |  |  |  |  |  |  | 8 | 9 | 10 | 7 |
|  |  | # of months (excluding zero entries) |  |  |  |  |  |  |  |  | 4 | 5 | 6 | 2 |
|  |  | Average NMR (zero-excluded) |  |  |  |  |  |  |  |  | 25 | 21 | 21 | 33 |
|  | **Kiziba** | Average camp population |  |  | 18,360 | 18,548 | 18,830 | 18,916 | 17,717 | 16,245 | 16,442 | 16,758 | 17,241 | 17,170 |
|  |  | Total Number of live births |  |  | 377 | 247 | 378 | 367 | 450 | 453 | 476 | 416 | 471 | 367 |
|  |  | # of months (including zero entries) |  |  | 8 | 6 | 12 | 12 | 12 | 12 | 12 | 12 | 12 | 9 |
|  |  | Average NMR (Zero-included) |  |  | 5 | 0 | 10 | 0 | 0 | 0 | 3 | 0 | 0 | 5 |
|  |  | # of months (excluding zero entries) |  |  | 2 |  | 3 |  |  |  | 1 |  |  | 2 |
|  |  | Average NMR (zero-excluded) |  |  | 20 |  | 31 |  |  |  | 32 |  |  | 24 |
|  | **Mahama** | Average camp population |  |  |  |  |  |  |  |  |  | 38,185 | 49,396 | 53,620 |
|  |  | Total Number of live births |  |  |  |  |  |  |  |  |  | 791 | 1,979 | 1,651 |
|  |  | # of months (including zero entries) |  |  |  |  |  |  |  |  |  | 8 | 12 | 9 |
|  |  | Average NMR (Zero-included) |  |  |  |  |  |  |  |  |  | 12 | 3 | 11 |
|  |  | # of months (excluding zero entries) |  |  |  |  |  |  |  |  |  | 3 | 5 | 7 |
|  |  | Average NMR (zero-excluded) |  |  |  |  |  |  |  |  |  | 31 | 8 | 14 |
|  | **Mugombwa** | Average camp population |  |  |  |  |  |  |  |  |  | 7,842 | 8,654 | 8,918 |
|  |  | Total Number of live births |  |  |  |  |  |  |  |  |  | 235 | 273 | 259 |
|  |  | # of months (including zero entries) |  |  |  |  |  |  |  |  |  | 9 | 11 | 9 |
|  |  | Average NMR (Zero-included) |  |  |  |  |  |  |  |  |  | 21 | 6 | 3 |
|  |  | # of months (excluding zero entries) |  |  |  |  |  |  |  |  |  | 5 | 2 | 1 |
|  |  | Average NMR (zero-excluded) |  |  |  |  |  |  |  |  |  | 38 | 35 | 25 |
|  | **Nyabiheke** | Average camp population |  |  | 13,515 | 14,183 | 14,345 | 14,997 | 13,767 | 13,007 | 14,364 | 14,032 | 14,133 | 14,383 |
|  |  | Total Number of live births |  |  | 177 | 192 | 241 | 310 | 250 | 333 | 329 | 362 | 398 | 280 |
|  |  | # of months (including zero entries) |  |  | 6 | 7 | 8 | 11 | 10 | 12 | 12 | 12 | 12 | 9 |
|  |  | Average NMR (Zero-included) |  |  | 24 | 8 | 0 | 10 | 17 | 15 | 8 | 5 | 3 | 7 |
|  |  | # of months (excluding zero entries) |  |  | 4 | 1 |  | 2 | 4 | 5 | 2 | 1 | 1 | 2 |
|  |  | Average NMR (zero-excluded) |  |  | 36 | 53 |  | 54 | 43 | 37 | 46 | 59 | 37 | 31 |
| **South Sudan** | **Adjoung Thok** | Average camp population |  |  |  |  |  |  |  |  |  |  | 39,200 | 37,227 |
|  |  | Total Number of live births |  |  |  |  |  |  |  |  |  |  | 860 | 696 |
|  |  | # of months (including zero entries) |  |  |  |  |  |  |  |  |  |  | 12 | 8 |
|  |  | Average NMR (Zero-included) |  |  |  |  |  |  |  |  |  |  | 2 | 2 |
|  |  | # of months (excluding zero entries) |  |  |  |  |  |  |  |  |  |  | 2 | 1 |
|  |  | Average NMR (zero-excluded) |  |  |  |  |  |  |  |  |  |  | 14 | 14 |
|  | **Bunj Hospital** | Average camp population |  |  |  |  |  |  |  |  |  |  | 191,958 | 191,958 |
|  |  | Total Number of live births |  |  |  |  |  |  |  |  |  |  | 875 | 305 |
|  |  | # of months (including zero entries) |  |  |  |  |  |  |  |  |  |  | 8 | 6 |
|  |  | Average NMR (Zero-included) |  |  |  |  |  |  |  |  |  |  | 28 | 7 |
|  |  | # of months (excluding zero entries) |  |  |  |  |  |  |  |  |  |  | 6 | 2 |
|  |  | Average NMR (zero-excluded) |  |  |  |  |  |  |  |  |  |  | 37 | 20 |
|  | **Doro** | Average camp population |  |  |  |  |  |  |  | 45,474 |  | 50,500 | 51,195 | 53,820 |
|  |  | Total Number of live births |  |  |  |  |  |  |  | 747 |  | 2,021 | 2,110 | 1,170 |
|  |  | # of months (including zero entries) |  |  |  |  |  |  |  | 6 |  | 12 | 12 | 7 |
|  |  | Average NMR (Zero-included) |  |  |  |  |  |  |  | 3 |  | 6 | 3 | 1 |
|  |  | # of months (excluding zero entries) |  |  |  |  |  |  |  | 1 |  | 6 | 4 | 1 |
|  |  | Average NMR (zero-excluded) |  |  |  |  |  |  |  | 21 |  | 11 | 8 | 6 |
|  | **Gendrassa** | Average camp population |  |  |  |  |  |  |  |  | 17,574 | 17,974 | 17,399 | 17,590 |
|  |  | Total Number of live births |  |  |  |  |  |  |  |  | 384 | 525 | 364 | 278 |
|  |  | # of months (including zero entries) |  |  |  |  |  |  |  |  | 9 | 12 | 10 | 8 |
|  |  | Average NMR (Zero-included) |  |  |  |  |  |  |  |  | 0 | 0 | 0 | 2 |
|  |  | # of months (excluding zero entries) |  |  |  |  |  |  |  |  |  |  |  | 1 |
|  |  | Average NMR (zero-excluded) |  |  |  |  |  |  |  |  |  |  |  | 19 |
|  | **Gentil Hospital** | Average camp population |  |  |  |  |  |  |  |  |  |  |  | 58,968 |
|  |  | Total Number of live births |  |  |  |  |  |  |  |  |  |  |  | 802 |
|  |  | # of months (including zero entries) |  |  |  |  |  |  |  |  |  |  |  | 7 |
|  |  | Average NMR (Zero-included) |  |  |  |  |  |  |  |  |  |  |  | 4 |
|  |  | # of months (excluding zero entries) |  |  |  |  |  |  |  |  |  |  |  | 1 |
|  |  | Average NMR (zero-excluded) |  |  |  |  |  |  |  |  |  |  |  | 25 |
|  | **Gorom** | Average camp population |  |  |  |  |  |  |  |  | 2,486 |  |  |  |
|  |  | Total Number of live births |  |  |  |  |  |  |  |  | 1,290 |  |  |  |
|  |  | # of months (including zero entries) |  |  |  |  |  |  |  |  | 12 |  |  |  |
|  |  | Average NMR (Zero-included) |  |  |  |  |  |  |  |  | 0 |  |  |  |
|  |  | # of months (excluding zero entries) |  |  |  |  |  |  |  |  |  |  |  |  |
|  |  | Average NMR (zero-excluded) |  |  |  |  |  |  |  |  |  |  |  |  |
|  | **Kaya** | Average camp population |  |  |  |  |  |  |  |  |  | 22,066 | 23,489 | 24,737 |
|  |  | Total Number of live births |  |  |  |  |  |  |  |  |  | 825 | 682 | 513 |
|  |  | # of months (including zero entries) |  |  |  |  |  |  |  |  |  | 12 | 12 | 7 |
|  |  | Average NMR (Zero-included) |  |  |  |  |  |  |  |  |  | 4 | 8 | 9 |
|  |  | # of months (excluding zero entries) |  |  |  |  |  |  |  |  |  | 3 | 3 | 2 |
|  |  | Average NMR (zero-excluded) |  |  |  |  |  |  |  |  |  | 16 | 33 | 30 |
|  | **Lasu** | Average camp population |  |  |  |  |  |  |  | 7,794 | 7,530 | 8,622 | 10,417 |  |
|  |  | Total Number of live births |  |  |  |  |  |  |  | 233 | 272 | 283 | 131 |  |
|  |  | # of months (including zero entries) |  |  |  |  |  |  |  | 10 | 11 | 12 | 6 |  |
|  |  | Average NMR (Zero-included) |  |  |  |  |  |  |  | 5 | 0 | 9 | 31 |  |
|  |  | # of months (excluding zero entries) |  |  |  |  |  |  |  | 1 |  | 2 | 3 |  |
|  |  | Average NMR (zero-excluded) |  |  |  |  |  |  |  | 53 |  | 55 | 63 |  |
|  | **Pamir** | Average camp population |  |  |  |  |  |  |  |  |  |  |  | 11,636 |
|  |  | Total Number of live births |  |  |  |  |  |  |  |  |  |  |  | 340 |
|  |  | # of months (including zero entries) |  |  |  |  |  |  |  |  |  |  |  | 8 |
|  |  | Average NMR (Zero-included) |  |  |  |  |  |  |  |  |  |  |  | 0 |
|  |  | # of months (excluding zero entries) |  |  |  |  |  |  |  |  |  |  |  |  |
|  |  | Average NMR (zero-excluded) |  |  |  |  |  |  |  |  |  |  |  |  |
|  | **Panrieng** | Average camp population |  |  |  |  |  |  |  |  |  |  |  | 81,231 |
|  |  | Total Number of live births |  |  |  |  |  |  |  |  |  |  |  | 552 |
|  |  | # of months (including zero entries) |  |  |  |  |  |  |  |  |  |  |  | 8 |
|  |  | Average NMR (Zero-included) |  |  |  |  |  |  |  |  |  |  |  | 5 |
|  |  | # of months (excluding zero entries) |  |  |  |  |  |  |  |  |  |  |  | 2 |
|  |  | Average NMR (zero-excluded) |  |  |  |  |  |  |  |  |  |  |  | 18 |
|  | **Yida** | Average camp population |  |  |  |  |  |  |  | 70,425 | 70,867 | 69,947 | 63,438 | 54,636 |
|  |  | Total Number of live births |  |  |  |  |  |  |  | 2,120 | 3,696 | 2,781 | 2,057 | 1,321 |
|  |  | # of months (including zero entries) |  |  |  |  |  |  |  | 11 | 12 | 12 | 12 | 8 |
|  |  | Average NMR (Zero-included) |  |  |  |  |  |  |  | 21 | 8 | 4 | 2 | 0 |
|  |  | # of months (excluding zero entries) |  |  |  |  |  |  |  | 9 | 10 | 8 | 2 |  |
|  |  | Average NMR (zero-excluded) |  |  |  |  |  |  |  | 26 | 10 | 6 | 12 |  |
|  | **Yusuf Batil** | Average camp population |  |  |  |  |  |  |  |  | 39,622 | 40,253 | 39,954 | 41,451 |
|  |  | Total Number of live births |  |  |  |  |  |  |  |  | 2,083 | 727 | 923 | 600 |
|  |  | # of months (including zero entries) |  |  |  |  |  |  |  |  | 11 | 6 | 12 | 6 |
|  |  | Average NMR (Zero-included) |  |  |  |  |  |  |  |  | 15 | 40 | 22 | 31 |
|  |  | # of months (excluding zero entries) |  |  |  |  |  |  |  |  | 6 | 6 | 9 | 6 |
|  |  | Average NMR (zero-excluded) |  |  |  |  |  |  |  |  | 27 | 40 | 29 | 31 |
| **Sudan** | **Fau 5** | Average camp population |  |  |  |  |  |  |  | 1,339 |  |  |  |  |
|  |  | Total Number of live births |  |  |  |  |  |  |  | 832 |  |  |  |  |
|  |  | # of months (including zero entries) |  |  |  |  |  |  |  | 6 |  |  |  |  |
|  |  | Average NMR (Zero-included) |  |  |  |  |  |  |  | 0 |  |  |  |  |
|  |  | # of months (excluding zero entries) |  |  |  |  |  |  |  |  |  |  |  |  |
|  |  | Average NMR (zero-excluded) |  |  |  |  |  |  |  |  |  |  |  |  |
|  | **Girba** | Average camp population |  |  | 9,154 |  |  | 5,421 | 5,856 | 6,328 | 6,685 | 6,673 | 6,521 | 6,902 |
|  |  | Total Number of live births |  |  | 310 |  |  | 291 | 226 | 283 | 331 | 325 | 309 | 204 |
|  |  | # of months (including zero entries) |  |  | 7 |  |  | 12 | 8 | 10 | 12 | 12 | 12 | 9 |
|  |  | Average NMR (Zero-included) |  |  | 0 |  |  | 0 | 0 | 0 | 0 | 0 | 0 | 0 |
|  |  | # of months (excluding zero entries) |  |  |  |  |  |  |  |  |  |  |  |  |
|  |  | Average NMR (zero-excluded) |  |  |  |  |  |  |  |  |  |  |  |  |
|  | **Kilo 26** | Average camp population |  |  | 12,648 | 13,082 | 9,484 | 7,641 | 7,950 | 8,366 | 8,641 | 12,273 | 9,326 | 9,699 |
|  |  | Total Number of live births |  |  | 387 | 300 | 253 | 305 | 177 | 308 | 300 | 310 | 299 | 225 |
|  |  | # of months (including zero entries) |  |  | 11 | 11 | 10 | 12 | 7 | 11 | 11 | 12 | 11 | 9 |
|  |  | Average NMR (Zero-included) |  |  | 7 | 0 | 0 | 0 | 0 | 0 | 0 | 0 | 0 | 0 |
|  |  | # of months (excluding zero entries) |  |  | 1 |  |  |  |  |  |  |  |  |  |
|  |  | Average NMR (zero-excluded) |  |  | 74 |  |  |  |  |  |  |  |  |  |
|  | **Shagarab I II III** | Average camp population |  | 22,517 | 23,123 | 23,712 | 20,965 | 22,767 | 23,567 | 28,325 | 31,069 | 32,367 | 36,042 | 34,003 |
|  |  | Total Number of live births |  | 371 | 813 | 778 | 710 | 940 | 673 | 967 | 1,058 | 1,030 | 891 | 640 |
|  |  | # of months (including zero entries) |  | 6 | 12 | 12 | 11 | 12 | 8 | 12 | 12 | 12 | 12 | 9 |
|  |  | Average NMR (Zero-included) |  | 5 | 3 | 2 | 0 | 0 | 0 | 0 | 0 | 0 | 0 | 0 |
|  |  | # of months (excluding zero entries) |  | 2 | 1 | 1 |  |  |  |  |  |  |  |  |
|  |  | Average NMR (zero-excluded) |  | 15 | 30 | 21 |  |  |  |  |  |  |  |  |
|  | **Um Gargour** | Average camp population |  | 10055.5 | 14,088 | 10,505 | 10,328 | 10,601 |  | 11,382 | 11,430 | 12,725 | 12,758 | 12,971 |
|  |  | Total Number of live births |  | 144 | 319 | 292 | 224 | 320 |  | 285 | 361 | 237 | 317 | 229 |
|  |  | # of months (including zero entries) |  | 6 | 12 | 11 | 9 | 11 |  | 10 | 12 | 9 | 12 | 9 |
|  |  | Average NMR (Zero-included) |  | 0 | 0 | 0 | 0 | 0 |  | 0 | 0 | 0 | 0 | 0 |
|  |  | # of months (excluding zero entries) |  |  |  |  |  |  |  |  |  |  |  |  |
|  |  | Average NMR (zero-excluded) |  |  |  |  |  |  |  |  |  |  |  |  |
|  | **Wad Sharifey** | Average camp population |  |  | 32,324 | 32,606 | 17,158 | 17,294 | 17,670 | 18,154 | 18,465 | 19,550 | 20,029 | 20,439 |
|  |  | Total Number of live births |  |  | 443 | 335 | 404 | 483 | 343 | 591 | 614 | 577 | 574 | 437 |
|  |  | # of months (including zero entries) |  |  | 10 | 10 | 10 | 12 | 8 | 12 | 12 | 12 | 12 | 9 |
|  |  | Average NMR (Zero-included) |  |  | 10 | 17 | 23 | 9 | 18 | 20 | 22 | 21 | 22 | 22 |
|  |  | # of months (excluding zero entries) |  |  | 3 | 5 | 6 | 4 | 6 | 5 | 8 | 6 | 6 | 4 |
|  |  | Average NMR (zero-excluded) |  |  | 33 | 34 | 39 | 28 | 24 | 43 | 33 | 42 | 43 | 50 |
| **Tanzania** | **Kanembwa** | Average camp population | 13,961 | 12,360 |  |  |  |  |  |  |  |  |  |  |
|  |  | Total number of live births | 519 | 498 |  |  |  |  |  |  |  |  |  |  |
|  |  | # of months (including zero entries) | 12 | 11 |  |  |  |  |  |  |  |  |  |  |
|  |  | Average NMR (zero-included) | 0 | 2 |  |  |  |  |  |  |  |  |  |  |
|  |  | # of months (excluding zero entries) |  |  |  |  |  |  |  |  |  |  |  |  |
|  |  | Average NMR (zero-excluded) |  |  |  |  |  |  |  |  |  |  |  |  |
|  | **Lugufu** | Average camp population | 54,472 | 48,943 | 38,119 | 25,312 |  |  |  |  |  |  |  |  |
|  |  | Total number of live births | 2384 | 2154 | 1,685 | 773 |  |  |  |  |  |  |  |  |
|  |  | # of months (including zero entries) | 12 | 12 | 12 | 8 |  |  |  |  |  |  |  |  |
|  |  | Average NMR (zero-included) | 0 | 4.9825 | 7 | 3 |  |  |  |  |  |  |  |  |
|  |  | # of months (excluding zero entries) |  | 6 | 7 | 2 |  |  |  |  |  |  |  |  |
|  |  | Average NMR (zero-excluded) |  | 9.965 | 13 | 14 |  |  |  |  |  |  |  |  |
|  | **Lugufu II** | Average camp population | 27,165 | 16,086 |  |  |  |  |  |  |  |  |  |  |
|  |  | Total number of live births | 842 | 465 |  |  |  |  |  |  |  |  |  |  |
|  |  | # of months (including zero entries) | 12 | 8 |  |  |  |  |  |  |  |  |  |  |
|  |  | Average NMR (zero-included) | 0 | 2 |  |  |  |  |  |  |  |  |  |  |
|  |  | # of months (excluding zero entries) |  | 1 |  |  |  |  |  |  |  |  |  |  |
|  |  | Average NMR (zero-excluded) |  | 17.54 |  |  |  |  |  |  |  |  |  |  |
|  | **Lukole** | Average camp population | 45,305 | 33,383 | 15,108 |  |  |  |  |  |  |  |  |  |
|  |  | Total number of live births | 2,234 | 1,897 | 524 |  |  |  |  |  |  |  |  |  |
|  |  | # of months (including zero entries) | 12 | 12 | 6 |  |  |  |  |  |  |  |  |  |
|  |  | Average NMR (zero-included) | 9 | 4 | 0 |  |  |  |  |  |  |  |  |  |
|  |  | # of months (excluding zero entries) | 8 | 3 |  |  |  |  |  |  |  |  |  |  |
|  |  | Average NMR (zero-excluded) | 14 | 14 |  |  |  |  |  |  |  |  |  |  |
|  | **Mtabila** | Average camp population | 17,095 | 15,693 | 41,744 | 38,224 | 36,377 | 37,418 | 37,304 |  |  |  |  |  |
|  |  | Total number of live births | 631 | 659 | 1,797 | 1,753 | 1,538 | 1,676 | 1,451 |  |  |  |  |  |
|  |  | # of months (including zero entries) | 12 | 12 | 12 | 12 | 12 | 12 | 11 |  |  |  |  |  |
|  |  | Average NMR (zero-included) | 2 | 2 | 2 | 9 | 12 | 9 | 12 |  |  |  |  |  |
|  |  | # of months (excluding zero entries) | 1 | 1 | 3 | 7 | 9 | 9 | 7 |  |  |  |  |  |
|  |  | Average NMR (zero-excluded) | 22 | 21 | 7 | 15 | 16 | 12 | 18 |  |  |  |  |  |
|  | **Mtabila II** | Average camp population | 33,642 | 36,830 |  |  |  |  |  |  |  |  |  |  |
|  |  | Total number of live births | 1331 | 1655 |  |  |  |  |  |  |  |  |  |  |
|  |  | # of months (including zero entries) | 12 | 12 |  |  |  |  |  |  |  |  |  |  |
|  |  | Average NMR (zero-included) | 7 | 1 |  |  |  |  |  |  |  |  |  |  |
|  |  | # of months (excluding zero entries) | 5 | 2 |  |  |  |  |  |  |  |  |  |  |
|  |  | Average NMR (zero-excluded) | 11 | 7 |  |  |  |  |  |  |  |  |  |  |
|  | **Mtendeli** | Average camp population | 24438 |  |  |  |  |  |  |  |  |  | 28,257 | 51,713 |
|  |  | Total number of live births | 961 |  |  |  |  |  |  |  |  |  | 714 | 2,385 |
|  |  | # of months (including zero entries) | 12 |  |  |  |  |  |  |  |  |  | 6 | 9 |
|  |  | Average NMR (zero-included) | 2.9625 |  |  |  |  |  |  |  |  |  | 8 | 4 |
|  |  | # of months (excluding zero entries) | 2 |  |  |  |  |  |  |  |  |  | 1 | 6 |
|  |  | Average NMR (zero-excluded) | 18 |  |  |  |  |  |  |  |  |  | 47 | 6 |
|  | **Muyovosi** | Average camp population | 24,763 |  |  |  |  |  |  |  |  |  |  |  |
|  |  | Total number of live births | 895 |  |  |  |  |  |  |  |  |  |  |  |
|  |  | # of months (including zero entries) | 12 |  |  |  |  |  |  |  |  |  |  |  |
|  |  | Average NMR (zero-included) | 0 |  |  |  |  |  |  |  |  |  |  |  |
|  |  | # of months (excluding zero entries) |  |  |  |  |  |  |  |  |  |  |  |  |
|  |  | Average NMR (zero-excluded) |  |  |  |  |  |  |  |  |  |  |  |  |
|  | **Nduta** | Average camp population | 22,878 | 34,980 | 24,780 |  |  |  |  |  |  |  | 59,369 | 120,140 |
|  |  | Total number of live births | 979 | 1426 | 1,183 |  |  |  |  |  |  |  | 2,863 | 4,040 |
|  |  | # of months (including zero entries) | 12 | 12 | 11 |  |  |  |  |  |  |  | 12 | 7 |
|  |  | Average NMR (zero-included) | 10 | 9 | 5 |  |  |  |  |  |  |  | 31 | 11 |
|  |  | # of months (excluding zero entries) | 8 | 7 | 4 |  |  |  |  |  |  |  | 12 | 6 |
|  |  | Average NMR (zero-excluded) | 15 | 16 | 14 |  |  |  |  |  |  |  | 31 | 12 |
|  | **Nyarugusu** | Average camp population | 58,237 | 51383 | 50,190 | 46,885 | 60,249 | 62,396 | 65,181 | 68,227 | 63,859 | 117,560 | 137,038 | 143,698 |
|  |  | Total number of live births | 1989 | 1996 | 1,669 | 1,919 | 2,555 | 2,358 | 2,444 | 1,939 | 2,563 | 11,999 | 5,779 | 3,827 |
|  |  | # of months (including zero entries) | 12 | 12 | 12 | 12 | 12 | 12 | 12 | 11 | 12 | 12 | 11 | 8 |
|  |  | Average NMR (zero-included) | 2 | 8.0525 | 7 | 1 | 0 | 2 | 2 | 2 | 6 | 6 | 13 | 14 |
|  |  | # of months (excluding zero entries) | 4 | 6 | 8 | 1 |  | 3 | 4 | 4 | 8 | 10 | 11 | 8 |
|  |  | Average NMR (zero-excluded) | 7 | 15 | 11 | 7 |  | 8 | 6 | 5 | 9 | 7 | 13 | 14 |
| **Thailand** | **Ban Mai Nai Soi** | Average camp population |  |  | 19,830 | 17,692 | 15,323 | 14,993 | 13,731 | 12,772 | 11,909 | 11,666 | 10,864 |  |
|  |  | Total number of live births |  |  | 509 | 489 | 442 | 376 | 312 | 274 | 212 | 220 | 145 |  |
|  |  | # of months (including zero entries) |  |  | 12 | 12 | 12 | 12 | 12 | 12 | 10 | 10 | 7 |  |
|  |  | Average NMR (zero-included) |  |  | 5 | 4 | 6 | 9 | 3 | 3 | 14 | 0 | 12 |  |
|  |  | # of months (excluding zero entries) |  |  | 2 | 1 | 2 | 3 | 1 | 1 | 3 |  | 2 |  |
|  |  | Average NMR (zero-excluded) |  |  | 22 | 53 | 37 | 36 | 34 | 37 | 46 |  | 43 |  |
|  | **Mae La** | Average camp population |  |  | 43,635 | 43,613 | 43,723 | 44,308 | 45,502 | 45,163 | 43,102 | 40,686 | 39,957 | 40,015 |
|  |  | Total number of live births |  |  | 1,255 | 1,378 | 1,309 | 1,086 | 1,223 | 1,038 | 857 | 979 | 819 | 456 |
|  |  | # of months (including zero entries) |  |  | 12 | 12 | 12 | 12 | 12 | 12 | 12 | 12 | 12 | 9 |
|  |  | Average NMR (zero-included) |  |  | 2 | 1 | 0 | 8 | 6 | 6 | 11 | 2 | 4 | 7 |
|  |  | # of months (excluding zero entries) |  |  | 2 | 2 |  | 6 | 4 | 4 | 6 | 2 | 2 | 3 |
|  |  | Average NMR (zero-excluded) |  |  | 12 | 9 |  | 16 | 18 | 19 | 23 | 9 | 24 | 20 |
|  | **Mae La Oon** | Average camp population |  |  | 16,279 | 16,953 | 17,128 | 16,354 | 15,947 | 14,440 | 12,996 | 10,979 | 10,536 | 10,091 |
|  |  | Total number of live births |  |  | 502 | 470 | 509 | 472 | 494 | 424 | 359 | 341 | 305 | 215 |
|  |  | # of months (including zero entries) |  |  | 12 | 12 | 12 | 12 | 12 | 12 | 12 | 11 | 12 | 9 |
|  |  | Average NMR (zero-included) |  |  | 16 | 10 | 2 | 13 | 13 | 13 | 5 | 17 | 15 | 9 |
|  |  | # of months (excluding zero entries) |  |  | 6 | 5 | 1 | 4 | 6 | 5 | 2 | 5 | 4 | 2 |
|  |  | Average NMR (zero-excluded) |  |  | 33 | 24 | 21 | 40 | 26 | 32 | 30 | 37 | 46 | 40 |
|  | **Mae Ra Ma Luang** | Average camp population |  |  | 16,967 | 17,721 | 18,441 | 17,550 | 16,397 | 15,273 | 14,089 | 12,481 | 11,265 | 10,392 |
|  |  | Total number of live births |  |  | 443 | 508 | 532 | 513 | 497 | 411 | 374 | 346 | 305 | 219 |
|  |  | # of months (including zero entries) |  |  | 12 | 12 | 12 | 12 | 12 | 12 | 12 | 11 | 12 | 9 |
|  |  | Average NMR (zero-included) |  |  | 13 | 14 | 12 | 8 | 14 | 9 | 15 | 23 | 14 | 11 |
|  |  | # of months (excluding zero entries) |  |  | 4 | 6 | 5 | 4 | 5 | 2 | 5 | 6 | 3 | 2 |
|  |  | Average NMR (zero-excluded) |  |  | 39 | 28 | 28 | 25 | 34 | 56 | 37 | 41 | 58 | 48 |
|  | **Nu Poh** | Average camp population |  |  | 18,032 | 17,876 | 17,628 | 17,683 | 17,552 | 15,803 | 13,722 | 12,588 | 10,407 | 8,378 |
|  |  | Total number of live births |  |  | 486 | 486 | 447 | 423 | 370 | 315 | 303 | 270 | 201 | 152 |
|  |  | # of months (including zero entries) |  |  | 12 | 12 | 12 | 12 | 12 | 12 | 12 | 12 | 10 | 8 |
|  |  | Average NMR (zero-included) |  |  | 9 | 2 | 0 | 2 | 3 | 0 | 9 | 3 | 6 | 0 |
|  |  | # of months (excluding zero entries) |  |  | 3 | 1 |  | 1 | 1 |  | 2 | 1 | 1 |  |
|  |  | Average NMR (zero-excluded) |  |  | 36 | 24 |  | 26 | 37 |  | 52 | 40 | 63 |  |
|  | **Tham Hin** | Average camp population |  |  | 7,725 | 7,997 | 8,304 | 7,740 | 7,406 | 7,498 | 7,240 | 6,743 |  |  |
|  |  | Total number of live births |  |  | 300 | 243 | 224 | 219 | 206 | 207 | 176 | 159 |  |  |
|  |  | # of months (including zero entries) |  |  | 11 | 10 | 10 | 10 | 10 | 10 | 9 | 9 |  |  |
|  |  | Average NMR (zero-included) |  |  | 7 | 12 | 8 | 0 | 4 | 12 | 16 | 0 |  |  |
|  |  | # of months (excluding zero entries) |  |  | 2 | 3 | 2 |  | 1 | 2 | 3 |  |  |  |
|  |  | Average NMR (zero-excluded) |  |  | 39 | 39 | 42 |  | 37 | 60 | 48 |  |  |  |
|  | **Umpiem Mai** | Average camp population |  |  | 18,293 | 17,681 | 17,830 | 17,784 | 16,296 | 14,067 | 12,501 | 11,822 | 11,117 | 10,741 |
|  |  | Total number of live births |  |  | 514 | 515 | 458 | 457 | 438 | 363 | 313 | 314 | 262 | 174 |
|  |  | # of months (including zero entries) |  |  | 12 | 12 | 12 | 12 | 12 | 12 | 12 | 12 | 12 | 8 |
|  |  | Average NMR (zero-included) |  |  | 24 | 9 | 2 | 2 | 0 | 0 | 9 | 7 | 3 | 16 |
|  |  | # of months (excluding zero entries) |  |  | 7 | 3 | 1 | 1 |  |  | 3 | 2 | 1 | 3 |
|  |  | Average NMR (zero-excluded) |  |  | 41 | 36 | 21 | 20 |  |  | 38 | 39 | 34 | 41 |
| **Uganda** | **Adjumani** | Average camp population |  | 52,870 | 32,188 | 13,445 |  |  |  |  | 79,002 | 103,710 | 124,328 | 208,986 |
|  |  | Total number of live births |  | 567 | 685 | 166 |  |  |  |  | 371 | 562 | 2,024 | 1,951 |
|  |  | # of months (including zero entries) |  | 7 | 12 | 6 |  |  |  |  | 10 | 12 | 12 | 9 |
|  |  | Average NMR (zero-included) |  | 3 | 1 | 0 |  |  |  |  | 5 | 11 | 9 | 3 |
|  |  | # of months (excluding zero entries) |  | 2 | 1 |  |  |  |  |  | 2 | 2 | 3 | 5 |
|  |  | Average NMR (zero-excluded) |  | 11.03 | 13 |  |  |  |  |  | 27 | 47 | 30 | 6 |
|  | **Bidibidi** | Average camp population |  |  |  |  |  |  |  |  |  |  |  | 272,206 |
|  |  | Total number of live births |  |  |  |  |  |  |  |  |  |  |  | 2,956 |
|  |  | # of months (including zero entries) |  |  |  |  |  |  |  |  |  |  |  | 9 |
|  |  | Average NMR (zero-included) |  |  |  |  |  |  |  |  |  |  |  | 13 |
|  |  | # of months (excluding zero entries) |  |  |  |  |  |  |  |  |  |  |  | 9 |
|  |  | Average NMR (zero-excluded) |  |  |  |  |  |  |  |  |  |  |  | 13 |
|  | **Imvepi** | Average camp population |  |  |  |  |  |  |  |  |  |  |  | 112,260 |
|  |  | Total number of live births |  |  |  |  |  |  |  |  |  |  |  | 442 |
|  |  | # of months (including zero entries) |  |  |  |  |  |  |  |  |  |  |  | 6 |
|  |  | Average NMR (zero-included) |  |  |  |  |  |  |  |  |  |  |  | 14 |
|  |  | # of months (excluding zero entries) |  |  |  |  |  |  |  |  |  |  |  | 3 |
|  |  | Average NMR (zero-excluded) |  |  |  |  |  |  |  |  |  |  |  | 28 |
|  | **Kiryandongo** | Average camp population |  | 140,73 |  |  |  |  |  |  |  | 41,118 | 58,282 | 52,228 |
|  |  | Total number of live births |  | 154 |  |  |  |  |  |  |  | 488 | 806 | 434 |
|  |  | # of months (including zero entries) |  | 6 |  |  |  |  |  |  |  | 12 | 12 | 8 |
|  |  | Average NMR (zero-included) |  | 13 |  |  |  |  |  |  |  | 3 | 0 | 7 |
|  |  | # of months (excluding zero entries) |  | 2 |  |  |  |  |  |  |  | 2 |  | 2 |
|  |  | Average NMR (zero-excluded) |  | 40 |  |  |  |  |  |  |  | 20 |  | 27 |
|  | **Kyaka II** | Average camp population |  | 17,390 | 15,039 | 15,442 | 16,917 | 15,026 | 16,651 | 19,323 | 22,843 | 23,646 | 27,216 | 25,373 |
|  |  | Total number of live births |  | 934 | 630 | 734 | 663 | 397 | 565 | 608 | 782 | 797 | 752 | 737 |
|  |  | # of months (including zero entries) |  | 7 | 11 | 12 | 12 | 12 | 12 | 12 | 12 | 12 | 12 | 9 |
|  |  | Average NMR (zero-included) |  | 0.68 | 5 | 0 | 0 | 0 | 0 | 7 | 0 | 0 | 0 | 0 |
|  |  | # of months (excluding zero entries) |  | 1 | 2 |  |  |  |  | 3 |  |  |  |  |
|  |  | Average NMR (zero-excluded) |  | 4.76 | 28 |  |  |  |  | 29 |  |  |  |  |
|  | **Kyangwali** | Average camp population |  | 19,552 | 15,803 | 19,189 | 19,882 | 19,167 | 20,735 | 26,051 | 39,592 | 40,860 | 41,671 | 43,439 |
|  |  | Total number of live births |  | 268 | 327 | 764 | 891 | 847 | 871 | 1,035 | 1,271 | 1,220 | 1,194 | 1,115 |
|  |  | # of months (including zero entries) |  | 6 | 12 | 12 | 12 | 12 | 12 | 12 | 12 | 12 | 12 | 9 |
|  |  | Average NMR (zero-included) |  | 0 | 4 | 1 | 2 | 7 | 4 | 22 | 21 | 7 | 9 | 9 |
|  |  | # of months (excluding zero entries) |  |  | 1 | 1 | 1 | 5 | 3 | 11 | 10 | 7 | 7 | 7 |
|  |  | Average NMR (zero-excluded) |  |  | 43 | 17 | 22 | 18 | 17 | 24 | 26 | 12 | 15 | 12 |
|  | **Nakivale** | Average camp population |  | 27,360 | 31,279 | 61,957 | 54,287 | 58,425 | 61,436 | 64,638 | 61,273 | 65,205 | 67,888 | 69,835 |
|  |  | Total number of live births |  | 608 | 1,516 | 1,552 | 1,873 | 2,009 | 2,099 | 2,286 | 1,820 | 2,156 | 2,671 | 2,133 |
|  |  | # of months (including zero entries) |  | 7 | 12 | 12 | 12 | 12 | 11 | 12 | 12 | 12 | 12 | 9 |
|  |  | Average NMR (zero-included) |  | 6 | 4 | 3 | 3 | 0 | 0 | 0 | 0 | 2 | 0 | 3 |
|  |  | # of months (excluding zero entries) |  |  | 4 | 3 | 4 |  |  | 1 |  | 2 |  | 4 |
|  |  | Average NMR (zero-excluded) |  |  | 10 | 13 | 9 |  |  | 5 |  | 14 |  | 8 |
|  | **Nyakabande** | Average camp population |  |  |  |  |  |  | 7,624 |  |  |  |  |  |
|  |  | Total number of live births |  |  |  |  |  |  | 269 |  |  |  |  |  |
|  |  | # of months (including zero entries) |  |  |  |  |  |  | 9 |  |  |  |  |  |
|  |  | Average NMR (zero-included) |  |  |  |  |  |  | 0 |  |  |  |  |  |
|  |  | # of months (excluding zero entries) |  |  |  |  |  |  |  |  |  |  |  |  |
|  |  | Average NMR (zero-excluded) |  |  |  |  |  |  |  |  |  |  |  |  |
|  | **Oruchinga** | Average camp population |  |  |  |  |  |  | 6,122 |  |  |  | 6,333 |  |
|  |  | Total number of live births |  |  |  |  |  |  | 209 |  |  |  | 229 |  |
|  |  | # of months (including zero entries) |  |  |  |  |  |  | 10 |  |  |  | 10 |  |
|  |  | Average NMR (zero-included) |  |  |  |  |  |  | 0 |  |  |  | 0 |  |
|  |  | # of months (excluding zero entries) |  |  |  |  |  |  |  |  |  |  |  |  |
|  |  | Average NMR (zero-excluded) |  |  |  |  |  |  |  |  |  |  |  |  |
|  | **Palorinya** | Average camp population |  | 28222 | 17,098 |  |  |  |  |  |  |  |  | 141,208 |
|  |  | Total number of live births |  | 222 | 354 |  |  |  |  |  |  |  |  | 1,808 |
|  |  | # of months (including zero entries) |  | 6 | 11 |  |  |  |  |  |  |  |  | 9 |
|  |  | Average NMR (zero-included) |  | 4 | 6 |  |  |  |  |  |  |  |  | 8 |
|  |  | # of months (excluding zero entries) |  | 1 | 2 |  |  |  |  |  |  |  |  | 5 |
|  |  | Average NMR (zero-excluded) |  | 25 | 33 |  |  |  |  |  |  |  |  | 11 |
|  | **Rhino Camp** | Average camp population |  |  |  |  |  |  |  |  | 16,828 | 21,083 | 37,466 | 90,983 |
|  |  | Total number of live births |  |  |  |  |  |  |  |  | 274 | 242 | 439 | 563 |
|  |  | # of months (including zero entries) |  |  |  |  |  |  |  |  | 10 | 11 | 11 | 9 |
|  |  | Average NMR (zero-included) |  |  |  |  |  |  |  |  | 0 | 0 | 3 | 0 |
|  |  | # of months (excluding zero entries) |  |  |  |  |  |  |  |  |  |  | 1 |  |
|  |  | Average NMR (zero-excluded) |  |  |  |  |  |  |  |  |  |  | 38 |  |
|  | **Rwamwanja** | Average camp population |  |  |  |  |  |  | 22,530 | 41,729 | 55,080 | 56,592 | 60,764 | 63,751 |
|  |  | Total number of live births |  |  |  |  |  |  | 416 | 1,475 | 1,438 | 1,656 | 2,106 | 1,810 |
|  |  | # of months (including zero entries) |  |  |  |  |  |  | 7 | 12 | 12 | 12 | 12 | 9 |
|  |  | Average NMR (zero-included) |  |  |  |  |  |  | 0 | 8 | 2 | 7 | 1 | 4 |
|  |  | # of months (excluding zero entries) |  |  |  |  |  |  |  | 6 | 1 | 4 | 1 | 4 |
|  |  | Average NMR (zero-excluded) |  |  |  |  |  |  |  | 16 | 20 | 21 | 17 | 8 |
| **Yemen** | **Basateen** | Average camp population |  |  |  | 14,992 | 15,978 | 21,770 | 21,112 | 22,606 | 23,120 |  |  | 29,662 |
|  |  | Total number of live births |  |  |  | 243 | 421 | 706 | 569 | 569 | 473 |  |  | 215 |
|  |  | # of months (including zero entries) |  |  |  | 7 | 12 | 12 | 11 | 12 | 10 |  |  | 7 |
|  |  | Average NMR (zero-included) |  |  |  | 0 | 21 | 0 | 8 | 7 | 11 |  |  | 5 |
|  |  | # of months (excluding zero entries) |  |  |  |  | 6 |  | 4 | 3 | 4 |  |  | 1 |
|  |  | Average NMR (zero-excluded) |  |  |  |  | 42 |  | 23 | 27 | 29 |  |  | 37 |
|  | **Kharaz** | Average camp population |  |  | 11,394 | 12,663 | 12,853 | 13,597 | 15,843 | 15,803 | 15,813 | 15,820 | 15,926 | 16,134 |
|  |  | Total number of live births |  |  | 209 | 389 | 524 | 594 | 539 | 550 | 473 | 476 | 286 | 186 |
|  |  | # of months (including zero entries) |  |  | 7 | 9 | 12 | 12 | 11 | 11 | 12 | 11 | 10 | 8 |
|  |  | Average NMR (zero-included) |  |  | 22 | 18 | 14 | 6 | 0 | 0 | 0 | 0 | 0 | 5 |
|  |  | # of months (excluding zero entries) |  |  | 3 | 4 | 5 | 4 |  |  |  |  |  | 1 |
|  |  | Average NMR (zero-excluded) |  |  | 51 | 41 | 34 | 19 |  |  |  |  |  | 43 |
|  | **Sanaa** | Average camp population |  |  |  | 22,960 | 23,053 | 23,359 | 36,346 | 36,709 |  | 8,795 | 8,271 |  |
|  |  | Total number of live births |  |  |  | 401 | 304 | 450 | 453 | 346 |  | 196 | 227 |  |
|  |  | # of months (including zero entries) |  |  |  | 9 | 12 | 12 | 11 | 10 |  | 8 | 9 |  |
|  |  | Average NMR (zero-included) |  |  |  | 0 | 12 | 9 | 12 | 0 |  | 21 | 0 |  |
|  |  | # of months (excluding zero entries) |  |  |  |  | 3 | 3 | 2 |  |  | 3 |  |  |
|  |  | Average NMR (zero-excluded) |  |  |  |  | 47 | 38 | 65 |  |  | 55 |  |  |
| **Zambia** | **Maheba** | Average camp population |  |  |  | 15,567 | 15,065 | 17,918 |  |  | 17,682 | 16,501 | 10,286 | 21,740 |
|  |  | Total number of live births |  |  |  | 2,616 | 498 | 538 |  |  | 356 | 300 | 279 | 197 |
|  |  | # of months (including zero entries) |  |  |  | 10 | 9 | 12 |  |  | 11 | 12 | 11 | 7 |
|  |  | Average NMR (zero-included) |  |  |  | 0 | 5 | 0 |  |  | 0 | 0 | 0 | 0 |
|  |  | # of months (excluding zero entries) |  |  |  |  | 1 |  |  |  |  |  |  |  |
|  |  | Average NMR (zero-excluded) |  |  |  |  | 47 |  |  |  |  |  |  |  |
|  | **Mayukwayukwa** | Average camp population |  |  | 10,491 | 10,079 |  | 10,149 |  |  | 11,835 | 11,881 | 12,454 |  |
|  |  | Total number of live births |  |  | 179 | 264 |  | 411 |  |  | 266 | 278 | 200 |  |
|  |  | # of months (including zero entries) |  |  | 6 | 10 |  | 12 |  |  | 11 | 12 | 7 |  |
|  |  | Average NMR (zero-included) |  |  | 0 | 3 |  | 0 |  |  | 7 | 4 | 0 |  |
|  |  | # of months (excluding zero entries) |  |  |  | 1 |  |  |  |  | 1 | 1 |  |  |
|  |  | Average NMR (zero-excluded) |  |  |  | 34 |  |  |  |  | 45 | 50 |  |  |

**Key:** NMR <12 per 1,000 (SDG target), 12 < NMR <40 (UNHCR/SPHERE standard), NMR>40 (UNHCR/SPHERE standard)
