# Supplementary material for: Neonatal mortality burden and trends in UNHCR refugee camps, 2006–2017: a retrospective analysis
Source: BMC Public Health. 2021 Feb 22;21:390. doi: 10.1186/s12889-021-10343-5 (PMC7898433; doi:10.1186/s12889-021-10343-5)
Supplement: Supplementary file 3 — Additional file 3: Supplementary File 3. Neonatal mortality rates reported in UNHCR HIS and host population surveys by location and year, 2008–2016. [file 12889_2021_10343_MOESM3_ESM.docx]

**Supplementary File 3: Neonatal mortality rates reported in UNHCR health information system and host population surveys by location and year, 2008-2016**

| Country | Camp | Year | Refugee NMR (zero included) | Refugee NMR  (zero excluded) | Host division (region/province/state) NMR | Host country NMR |
| --- | --- | --- | --- | --- | --- | --- |
| Bangladesh | **Leda Site** | 2011 | 8 | 25 | 30 | 32 |
|  |  | 2014 | 15 | 30 | 34 | 28 |
|  | **Nayapara** | 2011 | 9 | 27 | 30 | 32 |
|  |  | 2014 | 3 | 34 | 34 | 28 |
| Chad | **Belom** | 2014 | 19 | 32 | 33 | 34 |
|  | **Dosseye** | 2014 | 4 | 24 | 51 | 34 |
|  | **Mile** | 2014 | 3 | 16 | 28 | 34 |
|  | **Moyo** | 2014 | 9 | **45** | **26** | 34 |
|  | **Oure Cassoni** | 2014 | 2 | 23 | 23 | 34 |
|  | **Touloum** | 2014 | 4 | 26 | 28 | 34 |
|  | **Treguine** | 2014 | 3 | 18 | 40 | 34 |
| Ethiopia | **Jewi** | 2016 | 1 | 12 | 36 | 29 |
|  | **Kule** | 2016 | 1 | 12 | 36 | 29 |
|  | **Mai Aini** | 2011 | 4 | 38 | 44 | 37 |
|  |  | 2016 | 6 | **50** | **34** | 29 |
|  | **Melkadida** | 2011 | 1 | 13 | 34 | 37 |
|  | **Sheder** | 2016 | 3 | 40 | 41 | 29 |
|  | **Sherkole** | 2016 | 7 | **40** | **35** | 29 |
|  | **Tierkidi** | 2016 | 2 | 8 | 36 | 29 |
| Kenya | **Dagahaley** | 2008 | 3 | 10 | 33 | 31 |
|  |  | 2014 | 10 | 10 | 24 | 22 |
|  | **Hagadera** | 2008 | 1 | 4 | 33 | 31 |
|  |  | 2014 | 5 | 9 | 24 | 22 |
|  | **Ifo** | 2008 | 1 | 5 | 33 | 31 |
|  |  | 2014 | 11 | 13 | 24 | 22 |
|  | **Ifo 2** | 2014 | 14 | 14 | 24 | 22 |
|  | **Kakuma** | 2008 | 3 | 10 | 30 | 31 |
|  |  | 2014 | 5 | 8 | 20 | 22 |
| Liberia | **Bahn** | 2013 | 24 | **42** | **16** | 26 |
| Nepal | **Beldangi** | 2014 | 7 | **42** |  | **23** |
|  | **Sanishare** | 2011 | 4 | **38** | **30** | 33 |
| Rwanda | **Gihembe** | 2014 | 12 | **29** | **23** | 20 |
|  | **Kigeme** | 2014 | 8 | 25 | 25 | 20 |
|  | **Kiziba** | 2010 | 10 | **31** | **27** | 27 |
|  |  | 2014 | 3 | **32** | **25** | 20 |
|  | **Nyabiheke** | 2014 | 8 | **46** | **22** | 20 |
| Sudan | **Wad Sharifey** | 2010 | 23 | **39** | **34** | 34 |
| Tanzania | **Mtabila** | 2010 | 12 | 16 | 20 | 26 |
|  | **Nyarugusu** | 2015 | 6 | 7 | 27 | 25 |
| Uganda | **Kyangwali** | 2011 | 7 | 18 | 31 | 27 |
| Yemen | **Basateen** | 2013 | 7 | **27** | **22** | 26 |

**Bold indicates higher refugee vs host country population NMR*
